# Part III. Clinical Review and Hospital Reports

**Published:** 1834-01-01

**Authors:** 


### Clinical Review.

I. Hôtel Dieu.

*(Clinique of M. Dupuytren.)*

### Hydatid Tumors of the Wrist.

Encysted tumors, containing a number of small hydatidic bodies, of the size of pear-seeds, form occasionally on the palmar aspect of the wrist, under the aponeurosis, which exists at this part, and among the sheaths of the flexor tendons. Their nature is not unfrequently mistaken, and troublesome consequences have occurred from an injudicious treatment.

*Case* 1. A man-servant, aged 30, having one of these tumors, was admitted into the hospital. It had existed for two years, and extended from about two inches above to the same distance below the wrist-joint. It was somewhat flattened on its surface, and felt like those large sub-pericranial wens, which used to be called 'talpæ;' only that instead of being uniform it swelled out at the two ends, and was girt tight about the middle, by the palmar ligament—thus resembling a double-pouched wallet. The skin at the part was not at all affected in colour. The hand could not be bent upon the forearm, and the movements of the fingers upon the hand were also impeded. Severe lancinating pains extended along the whole palmar extent of the forearm, and deprived the patient of sleep; they were not however increased by pressure upon the tumor; but when this was done, a sort of crepitation was perceptible, just as when we pat a leather pouch, containing some very small leaden bullets; besides, the movement of the small bodies from one end to the other could be felt in this way, and either end might be made to swell out by compression upon the other. Experience having shewn that any other mode of treatment but free incision of these tumors, or amputation of the forearm, (as some have recommended in all cases,) is not only useless but possibly very dangerous, it was determined in the present instance to cut fairly through the sac, empty its contents, and induce a suppurative granulation from its interior. The operation was performed thus: while an assistant pressed firmly upon the palmar ligament, so as to prevent the discharge of the fluid from the whole cyst, a transverse incision was made through the integuments and walls of one of its lobules, care being taken to avoid wounding the annular ligament of the wrist. An innumerable quantity of small, white, hard, oval, or rounded bodies, immediately escaped; the other lobule was then cut open, and a similar discharge flowed out. The sac was thus entirely emptied. A small portion of its walls was pulled out of the wounds, and snipped off with scissors;—it was found to be firm and fibrous, like wet parchment. A piece of lint was pushed into each orifice, so as to prevent them healing outwardly, and a light dressing laid over it. The strictest antiphlogistic treatment was enforced, the patient being bled, leeched, &c., and the arm kept suspended, and constantly wet with a cooling wash.

On the 3d day, the wounds were examined; their edges were so puffy and swollen, that the pieces of lint had been forced out, and the openings were almost closed; the hand and forearm were red and inflamed, and so exquisitely tender, that the slightest motion caused great pain. The lips of the wound were gently separated, and pressure made, so as to squeeze out any contained matter; the dossils of lint were then replaced, and the member enveloped in emollient fomentations.

Every unfavourable symptom gradually abated, granulations sprung up from the bottom, and on the fifteenth day the cure was assured. Great care was taken each day to empty the pouch of any pus which might be confined. Within the mouth the wounds were completely healed. The use of the local baths was ordered to be continued, for the purpose of relaxing the joint and facilitating its movements.

*Case* 2. A man, aged 29, had been annoyed with one of these tumors for about a twelvemonth; it was very similar to that in the preceding case. The operation has not yet been performed, [month of July.]

In regard to the frequency of these encysted tumors, there seems to be much discrepancy of opinion. Some surgeons tell us that they have never met with them; and yet Dupuytren has treated upwards of fifty cases in the last twenty-five years.

The inference is but too apparent, viz. their nature has not been properly understood. Authors have confounded them, sometimes with white-swellings, and at other times with hydrarthroses of the wrist, or steatomatous, or lipomatous tumors, treating them with resolvent applications, &c. &c. The diagnosis will be much facilitated by attending to the two following signs.

1. They invariably are seated on the palmar side of the wrist, under the carpal ligament, and extend upwards and downwards, forming a bilobulated swelling, like a double-pouched wallet.

2. They crepitate and communicate a feeling, as if little bodies passed and repassed, when pressure is made upon them. Sometimes indeed this symptom is rather obscure; but when it can be felt distinctly, it may be considered as truly and exclusively characteristic of these tumors.

As to the nature of these hydatidic bodies which are found in the sac, observers are not agreed; Dupuytren regards them as organized and living beings; Dumeril and other naturalists as productions which are altogether inorganic; some German physiologists attribute them to the agency of galvanic currents; Professor Petrunti of Naples thinks that they are detached aneurisms or varices of the lymphatic vessels; Dr. Rognetta inclines to the same opinion, and alludes to the occurrence of similar hydatidic tumors developed between the choroid coat and the retina. It is worthy of notice, that in the subject of the second case, mentioned above, there was a coexistent disease of the lymphatic vessels of the whole arm.

### Club-Foot in New-born Children.

A child, 15 days old, was brought to M. Dupuytren's consultation; there was an intro-version of the left foot; the right one being quite natural. A bent splint, well padded, was applied to the outer side of the leg, and a narrow roller then passed from the one to the other, so as to give the requisite inclination outwards. The cure is thus completed usually in four or six weeks.

It is a curious fact, that when a child is affected with this deformity in one foot only, the corresponding limb is generally shorter and worse-fed than the other; and that as soon as the foot recovers its proper position, the irregularity of the limb begins to be, and is soon rectified. Such is Dupuytren's experience.

### Encysted Tumors on the Head.

A woman, 30 years of age, presented a number of these tumors, scattered on different parts of the scalp; they varied in size, from that of a hen's egg, to that of a filbert, the largest ones being situated behind and on the sides. They caused great inconvenience, when the patient rested her head on the pillow. The integuments over some were inflamed and painful.

With regard to the method of extirpating these tumors, Dupuytren prefers that which has been called "enucleation;" an incision is made through the integuments—the cyst is then worked about, and detached from its surrounding connexions with a small fine spatula, passed round between these and the cyst; the tumor is thus quickly and neatly unkernelled, and started out from its socket. It is altogether a much more adroit, less tedious, less painful, and less dangerous method, than that of dissecting out the sac with the scalpel. When a tumor is very large, M. Dupuytren advises that a circular portion of the integuments be detached from the summit, and the operation then finished, as we have mentioned, with the spatula.

The reporter adds that he has occasionally observed, that the development of wens upon the head is somehow connected with pregnancy.

### Syphilis and Syphiloid Diseases.

*Case* 1.—A young female was admitted, for several chronic ulcers upon both legs; the two largest, each about three inches across, were situated on the outer side of the right limb. They had been, for the space of two years, treated as scrofulous sores, and almost every anti-strumous medicine tried without avail. They were indolent and reddish—the granulations were large and atonic—the edges were thin and separated, or, as it were, unglued, and the base or centre of the sores prominent above the rest of the surface. The patient was decidedly of a scrofulous habit. Dupuytren suspected that there was a venereal taint, but the young lady vowed that she was a maid. However, upon an examination, the very obvious traces of having been "unmaided" were not to be gainsayed; and his suspicions being confirmed by other circumstances, the antisyphilitic treatment of the Hôtel Dieu was ordered. [A pill, composed of 1-6th of a grain of corrosive sublimate, 1-4th of a grain of extract of opium, and two grains of extract of guiac, to be taken thrice a day, and also the decoction of sarsaparilla, with a spoonful or two of sudorific syrup; complete abstinence from wine, spirits, and coffee. This treatment is to be continued, in most cases, for two months.]

In twelve days a very visible amendment had taken place, and in twenty more the greater number of the sores were healed.

*Case* 2.—A woman, aged 40, had for eighteen months suffered under ophthalmia in both eyes; every now and then it became worse, and she was obliged to have the eyes constantly bound up, as the admission of the light was quite intolerable. The conjunctiva was found highly vascular, but not much swollen, and there was no discharge; the inner circle of the iris was somewhat injected, and the pupil half closed; the bottom of the eye could not be properly seen. Purgatives, blisters, setons, &c. had been repeatedly tried without success.

The great oculo-cerebral distress which this patient suffered, being much greater than one might expect from the visible local malady, it was supposed probable that the retina might be in a state of inflammation, and if so, the character of the inflammation was specific. The patient resolutely denied having ever incurred the risk of syphilitic infection; but, in spite of this, the mercurial treatment was decided upon, and if no improvement took place in a fortnight, it was to be laid aside. But before the expiry of that period, the ophthalmia had greatly diminished, so that she could bear the admission of light—in another fortnight she was cured. The patient afterwards confessed, that she once had syphilis in her youth.

*Case* 3.—A woman, aged 40, presented an ulcer, situated below the right mamma. It was from three to four inches across, and in some respects resembled a cancerous sore. It was deep, excavated, and filthy—its surface was covered with a putrid eschar or slough—the discharge was most fetid—the edges separated all round, and the adjacent integuments were œdematous. The patient suffered lancinating pains in the sore; the axillary glands, however, were not affected. The disease had commenced, eleven months ago, with a tumor, which subsequently broke; she had been treated for cancer. The antisyphilitic treatment was adopted; the chloride of lime wash was applied to the sore, in order to detach the adhering gangrenous detritus.

In three weeks, the sloughs were all removed, and the aspect of the sore in every respect so much improved, that under simple treatment it soon healed up.

*Case* 4.—A countrywoman from Picardy, aged 36, had laboured for ten months under complete amaurosis of both eyes. She was four months pregnant; but this circumstance was not confessed at the time of admission; the disease had supervened upon an obstinate ophthalmia. On examination, the eyes were found to be quite clear, the pupils insensible to the light, the size of the eyes natural; she complained of severe frontal cephalalgia, but of no pain in the affected organs. The patient was of an exceedingly nervous irritable constitution—the complexion had an earthy hue, and the breath was very fetid. The general functions seemed to be moderately sound. When questioned as to the possibility of venereal infection, she at once denied it. This being the case, and there being no sufficient grounds to infer a rational suspicion, the usual treatment of blistering, purging, vomiting, &c. was employed for some time, but with no benefit.

Again she was examined upon the subject of her previous ailments; and now she confessed having had syphilis, eleven months before. The antiphlogistic treatment was prescribed forthwith, and in ten days the sight of the left eye was completely recovered, and that of the right one much improved. The mercury began to act violently on the intestinal tube, and the consequence of this was, that abortion ensued. In the sequel, the eye-affection was effectually cured.

*Case* 5. A woman, 30 years of age, the mother of one child, and pregnant five months with a second, was admitted into the Hôtel Dieu for an enormous puffy swelling of the whole lower lip; it was red, painful, ulcerated and cracked: there were also three sores, each of the size of a forty-sous piece, on the left cheek, and others, which were smaller, close to the corresponding commissure of the lips. The patient did not exhibit any of the constitutional symptoms of syphilis. She had been six weeks at the Hôpital St. Louis, and treated there with repeated leechings, poultices, and a strict antiphlogistic regimen, but quite unsuccessfully. M. Dupuytren, considering the long standing of the disease, its obstinate resistance to all antiphlogistic remedies, and the filthy aspect of the sores, concluded that it was dependent upon a venereal taint, and ordered the antisyphilitic treatment. No decided amendment took place, till the twelfth day; when cicatrization commenced, and quickly advanced to a perfect cure. The topical application was calomel sprinkled upon the sores, and a mild dressing over it. The course of pregnancy was not at all interfered with by the mercurial treatment.

*Remarks*. Dr. Cayol, one of the able editors of the Revue Medicale, appends a few observations to the preceding report. He states, that although mercury has long been justly considered as a touch-stone to discover the syphilitic infection in obscure and doubtful cases, it is by no means either absolute or exclusive in this respect. It is not absolute, for experience has taught us that not only do some venereal cases resist its operation, but they are most unequivocally exasperated by it, under whatever form it be administered; neither is it exclusive, for many other diseases besides syphilis are speedily and effectually cured by its use.

Without alluding to tumors and chronic swellings, to many old and indolent ulcers, and a host of other maladies, we shall at present mention its very striking efficacy in some cases of abdominal effusion supervening upon peritonitis or enteritis. If the mercurial ointment be freely rubbed in upon the abdomen we have seen immense sero-purulent collections (eight to ten pints for example) dissipated in a few days.

The object of these remarks is to point out the incorrectness of the opinion adduced by the reporter of the preceding clinique, that when a disease which has resisted other modes of treatment, yields to the use of mercury, we should therefore regard it as depending upon a venereal taint.

The medical man who permits his mind to entertain such an idea will run the hazard of frequently compromising the honor and dignity of the profession by rash and groundless suspicions of the moral conduct of his patients.

---

### II. Hôpital du Midi.

[*Clinique of M. Ricord*]

### Hydatidic and Cancerous Disease of the Testicle.

A young man, aged 22, was admitted on the 14th February, 1833. Eleven months previously he had observed that his left testicle had increased considerably in weight, and that it was indurated at the base; he felt, however, no pain in it. From that time its size became greater and greater. He consulted several surgeons who told him that it was a simple hydrocele, unattended with any danger. But not being satisfied with their opinions he came up to Paris from the country to consult M. Ricord, who discovered, that in addition to the disease of the testicle, there was also a considerable tumor perceptible on the left side of the abdomen; he complained of severe pains in the region of the kidneys; and these he said had preceded for some days the appearance of the swelling in the abdomen; and of late he felt also prickings in the body of the testicle, which was now so much enlarged as to measure four inches in length, and three at its broadest part: at the inner side, next to the septum scroti, a small glandular body could be felt, of the size and consistence of a healthy testicle. The distended scrotum upon pressure was resistant; it was not however the resistance of fluctuation, but of a bag entirely filled with fluid. The spermatic cord was much stretched, and the vessels were separated from each other, and dispersed in a fan-like manner over the surface of the testicle. The abdominal swelling extended from the false ribs to the pelvis, and from the linea alba, outwardly to the flank. It offered a resistance upon being pressed, very similar to the resistance of the scrotum. Different opinions were still entertained as to the nature of these two tumors; M. Ricord deemed them to be carcinomatous, but thinking that the tunica vaginalis inclosed a good deal of fluid, he first punctured it with a cataract needle, before withdrawing which he turned it round about in different directions, but found no solid obstacle: a few drops of serosity flowed out from the wound. The result of this trial satisfied M. Ricord that there was fluid in the sac, and he reasoned that the interposition of this prevented any accurate examination of the testicle, which, in all probability, was organically diseased, considering the great weight of the swelling. On the 28th he introduced a trocar in two different places, but not a drop of fluid escaped.

The diagnosis now was that the tumor contained serous cysts, whose parietes were fragile and easily torn. If this supposition were correct, the same opinion might be extended to the tumor in the abdomen.

*March 4th.* One part of the swelling had become much more prominent, than the rest; and there, it gave a feeling of fluctuation. A straight bistoury was plunged into the depth of four lines, but no discharge followed, although still we thought that fluctuation was perceptible; a probe being now introduced, could be turned round in all directions with ease. Meanwhile the abdominal swelling had increased in bulk considerably, so as to cause oppression to the breathing and general disturbance of the health; and that of the scrotum had almost doubled its size; the wound made with the bistoury had thrown out a cancerous fungus, and the skin around had assumed an erysipelatous purplish aspect; an eschar, as large as a five-franc piece had formed on the most depending part; when this was detached, the subjacent cellular tissue was found sphacelated, and some black fœtid sanies flowed out. In this unfavorable state of things, every one was much surprised one day to find that the abdominal swelling had quite vanished; the parietes had recovered their normal level; the oppression had ceased as if by magic; pressure was no longer painful, and the patient felt within himself light and happy, at the prospect of his return to health, as he supposed. The bowels were twice moved freely, three or four hours after the subsidence of the tumor.

Upon the supposition that the abdominal affection was in a great measure removed, it became now a question, whether the operation of castration was advisable, as there were no doubts that the disease of the testicle, if left to itself, would ultimately prove fatal. The chances of recovery, in the present state of uncertainty, and in the precarious health of the patient, were however far from promising. The testicle was extirpated on the 25th, and for a few days subsequently, the patient continued in a favourable condition. On the 29th symptoms of fever set in, and the wound was pale, and crepitating at some points; from this period he gradually sunk, until death.

*Dissection.* The extirpated testicle was one third smaller than it had been before the operation, in consequence of a quantity of limpid serum having flowed out from an incision with the point of the bistoury. A deep longitudinal cut was made through its entire length; much transparent fluid, contained in serous cysts of a rounded form, and varying in size, from that of a pea, to that of a filbert, escaped. One of these cysts, which was as large as a fowl's egg, had been inadvertently opened during the extirpation of the mass. The rest of the tumor was made up of an encephaloid degeneration, in various degrees of progress; on the back part, there was a smaller tumor, of the size of a wallnut, in which the vas deferens terminated, and which contained this encephaloid matter, marbled with effusions of blood. The abdomen was now opened, and a large ovoid pouch, extending from the linea alba to the vertebral column, and from the edge of the ribs to the edge of the pelvis, so as to cover the descending colon and the left kidney, presented itself to view; this pouch had membranous walls, and was half filled with a fluid which obscurely undulated upon percussion. It adhered firmly to the psoas muscle, and also to the aorta and spine. While dissecting it out from its attachments, it burst, and a quantity of yellowish semifluid matter, having a strong fæcal smell, flowed out; a communicating aperture, two inches wide, existed between the cavity of the sac, and the duodenum; and there the volume of the intestine was considerably enlarged. On slitting open the sac in its whole length, the contents were found to be a greyish coloured fluid, seemingly composed of pus and chyme blended together. In conclusion, we may allude to the circumstance of the very sparing escape of fluid from the apertures made with the needle, and trocar, before the operation. Probably the cause of it was that the fluid became diffused through the cellular texture of the scrotum (which were indeed observed upon making the first incision, to be infiltrated with a gelatinous serosity) or in the meshes of the false membranes, forming the cysts.

[We are surprised that French authors, whose knowledge of pathology we have always pleasure in admitting, persist in applying the term cancerous to the encephaloid disease, and in designating all tumors of the testicle, whatever be their nature, by the general appellation of sarcocele. Such inaccuracies ought to be expunged.—*Tr.*]

### III. St. George's Hospital.

The cases contained in the following report, have occurred in the practice of Mr. Cæsar Hawkins. We are indebted to the kindness of that gentleman for a perusal of his hospital case-book, from which the particulars will be found to be extracted. Not having personally witnessed the facts, we cannot perhaps do adequate justice to the questions of importance or interest connected with them. But it is a matter of satisfaction to reflect, that the source from which they are obtained is an ample guarantee of their fidelity. At the present day this must be looked on as no contemptible recommendation.

This report will be necessarily miscellaneous in its charaeter.

DISEASES OF THE TESTIS.

1. *Chronic Inflammation of the Testis—Mercury.*

John Peascud, æt. 43, admitted November 21, 1832, under the care of Mr. Hawkins.

Left testicle enlarged—of a globular form—firmer at the lower than the upper portion—hard and knotty—with some fluid in the cavity of the tunic in front and above—vas deferens thickened. Lower part of the tumor tender to the touch—pain in the course of the cord.

Twelve months ago hernia humoralis on the right side; it passed away spontaneously in the course of a few days. Soon after this the left testis began to swell at its inferior part, and the enlargement gradually increased till two months ago, since which it has been stationary. Has done nothing for the complaint. General health good.

*Pil. hyd.* gr. v. *nocte maneque.*

27th. Tenderness of the lower part of the tumor increased.

*Hirud.* vj. *testi.*

*Dec.* 1. Pyrexia—headache; was first chilly last evening and perspired greatly.

*H. sennæ stat. Hs. salin. 6tis horis. Omr. pil.*

On the 3d, the pyrexia was gone, and the patient was reinstated in the ordinary diet. On the 4th the pills were directed to be resumed, but only at night. On the 9th they were ordered to be continued twice daily.

12th. Mouth sore. Testicle diminishing.

15th. Mouth no longer sore—testicle much smaller.

*Rep. pil. ter die.*

Jan. 5. Testis much smaller—less fluid—hardness still persists.

*Rep. pil. 4ter indies.*

*Jan.* 9. Left the hospital at his own desire. Cannot be considered as wholly cured.

CASE 2.—*Fungoid Disease—Castration—result undetermined.*

George Stedman, æt. 43, admitted Oct. 24, 1832.

Considerable enlargement of the left testis, which is nearly of a globular form, and of an uniform surface. The tunica vaginalis adheres to the testis throughout the greater part of its circumference, but across the upper third a distinction is perceptible, and the tunic can be felt above it. Tumor elastic in its structure, and apparently containing fluid. Cord not augmented in size. No tenderness on pressure of the testis—some pain in the back and in the direction of the cord.

Health tolerably good—pulse 76, and deficient in strength—bowels occasionally relaxed without appreciable cause.

Complaint began two years ago with swelling at the bottom of the scrotum, attended with some pain. Knows no cause for the disease. Has continued to follow his employment during this period, with the exception of one week only.

On the 30th, an explorer was introduced into the lower part of the tumor, but no fluid was discovered; its introduction into the upper part was followed by the flow of two or three ounces of a clear and almost colourless liquid. The swelling was now found to extend somewhat upwards behind the fluid contained within the tunic.

*Broth diet—confinement in bed.*

*Nov.* 7. The testis has diminished a little in size, and parts of it feel softer, and more like fluid; one soft small prominence at the lower part has become more obvious. A needle has been introduced into two or three places, but only a few drops of serum have escaped.

In consultation it has been decided that the testicle should be removed. After a preparatory dose of aperient medicine, this was done upon the 8th.

Two semi-elliptical incisions were made in the skin, and, the tumor being removed by dissection from the scrotum, the cord was held by an assistant whilst it was divided. Two arteries were separately tied in the cord, and some were secured in the cellular membrane. A strip of lint dipped in oil was introduced between the edges of the wound at its bottom, in order to prevent their too speedy re-union; some sutures were employed; and the wound dressed with plaister, compresses, and the T bandage. Very little blood was lost in the operation.

On making a section of the tumor, it presented a medullary structure, interspersed with portions of yellow lymph. Superiorly, the cavity of the tunica vaginalis contained several ounces of fluid. It appeared, on an accurate examination, that the testis, at the upper part of the tumor, was healthy, and expanded on its surface. With this healthy part of the testis, the hydrocele was connected. There were neither cysts nor bloody extravasations in the tumor.

3, p.m. *Trœ opii,* ♏xxx.

10, p.m. *Pulv. ip. c.* gr. x. *ex Hu. salin. stat.*

*H. salin.* c̄ *Pulv. ip. c.* gr. iv. 6*tis hor.*

9th. Slept little during the night—experiences some pain in the abdomen.

*Vesp. Trœ. opii,* ♏xxx.

10th. Pulse 130—tongue white, rather dry—bowels have not acted since the operation—feels easy.

*Hyd. sub.* c̄ *Op.* gr. j. *h. s. Ol. ric. cràs. Hs. salin.* 6*tis horis.*

11th. Much the same.

*To have some meat to-day.*

The relief from the improvement in the diet would appear to have been marked. No further symptoms of general disorder occurred in the subsequent progress of the case.

On the 12th, the wound was dressed. Two sutures were removed, and one ligature was taken away. Some little suppuration took place around the cord, but it was not of prejudicial importance.

On the 20th, the other ligatures were abstracted.

On the 11th December, the wound was cicatrized, with the exception of one place in the scrotum. At his own desire, he went into the country on the 12th.

CASE 3.—*Fungoid Disease—Castration—Result undetermined.*

George Bennett, æt. 23, admitted October 24th, 1832.

Testis of the right side enlarged, and the tumor of a pyriform shape—testicle not felt distinctly inferiorly and behind—fluctuation detected superiorly and in front. On standing or in walking, a sense of dragging experienced in the course of the cord. General health pretty good—bowels regularly open.

He first perceived the enlargement of the testicle seven months ago, from which period the tumor has progressively increased. Is not aware of any obvious cause.

On the 25th, the tumor was punctured with a trocar, but no fluid was obtained. On the 27th, a grooved needle was introduced in two places in the upper part of the tumor; a small quantity of fluid was discharged. On the 3d of November, it was determined to offer the patient the chance that mercury presented.

*Pil. hyd.* gr. v. *bis die.*

6th. The testis has a little increased in size, and whatever fluid may have covered it superficially has been absorbed. The spermatic cord appears to be healthy.

13th. Mouth rather sore. The fluid in the tunic has accumulated again.

*Pil. hyd.* gr. x. *nocte, et* gr. v. *mane.*

20th. Tumor would seem to have increased. Mouth not so much affected as it was.

*Aug. dos. ad* gr. x. *nocte maneque.*

24th. Tumor still on the increase.

*Dec. sars. c.* Oj. *quotid. Omr. Pil.*

On the 5th of Dec. a consultation was held, and on the 6th the testis was removed. The spermatic veins were varicose, and the cord slipped from the finger of the assistant. Some bleeding ensued, there was a consequent delay and trifling embarrassment, and the patient was removed to bed before the dressings were applied. Several arteries were afterwards secured, and the usual sutures, &c. had recourse to.

The testis was converted into a medullary structure, with a small quantity of yellow lymph. Some cells, holding serous fluid, were interspersed in its substance, and portions of coagulated blood were found there. No trace of the tubuli testis remained, and the tunica albuginea appeared to afford an investment to the disease. The tunica vaginalis contained no fluid.

*Tr. opii,* ♏xx. *ult. nocte.*

7th. Some headache—tongue furred—pulse 106. Some swelling in the cord and its vicinity, with an oozing of serum from the wound.

*H. sal.* c̄ *Vin. ant. t.* ♏xv. *6tis hor.*

8th. Less pyrexia and functional disturbance. Troublesome cough. Discharge of bloody serum from the wound.

*Adde Hui. oxymel. scill.* ʒj.

9th. Pulse 120—tongue furred—bowels twice opened—slight shivering—countenance flushed—a blush of redness near the wound.

The sutures were removed, and a ligature came away. Some putrid bloody pus escaped.

*Beef-tea,* ℔j.

10th. Better. The swelling of the wound is less—the redness has disappeared.

We need not pursue the diurnal details. Healthy suppuration ensued, granulation succeeded, a sinus which remained for some time filled up, and the patient was discharged on the 29th of January.

The results of the operation in both these cases would be gratifying, if candour would permit us to form a decisive opinion, after the lapse of so brief a period. Unhappily, castration for fungoid disease of the testis is rarely attended with success. The instances which we have witnessed at this hospital within the last six or seven years have been discouraging to a great degree. The late Mr. Rose removed a testis affected with this malady, and the disease soon appeared in the lumbar glands. The same thing was observed in a patient of Mr. Keate's. In another patient there appeared a tumor in the neck. Mr. Brodie amputated a testicle affected with hydatid disease. The patient died of peritonitis, and a similar alteration was found in the lymphatic glands of the loins. We might mention other cases equally calculated to display the unpromising nature of the operation. The persons whose cases are contained in this report received strict injunctions, when they left the hospital, to return if they discovered a reappearance of disease. Perhaps their absence may be looked upon as evidence of their not having suffered a relapse.

CASE 4.—*Doubtful Affection of the Testis—Cure.*

Joseph Bryony, æt. 13, admitted March 13, 1833.

Has apparently a small tumor of the left epididymis, painful on pressure, but not so when he is quiet.

Four months ago he had pain and swelling in the course of the left spermatic cord and epididymis. These symptoms disappeared in a few days without medical treatment. One week ago had a similar attack, ending in the present condition of the parts.

*Lot. spt. testi.*

*Infus. cascarill.* ℥j. c̄. *Liq. potass.* ♏. xv. *ter die.*

Under this treatment the swelling subsided, and the health, if impaired, became restored. He was therefore dismissed cured.

Mr. Hawkins was inclined to think this an instance of scrofulous disease, and the benefit derived from the treatment employed would probably favour the idea.

### REMOVAL BY OPERATION OF A CICATRIX FROM A BURN.

*Case.* Jane Perrin, æt. 10, admitted March 13, 1833.

One year ago this child was burnt on the anterior aspect of the elbow-joint. The burn was nearly healed without contraction, but as soon as the cicatrix was almost formed it gradually began to contract.

The cicatrix now covers the inner and fore-part of the joint, extending over two-thirds of the arm and about as much of the forearm. The line of contraction is nearly in the direction of the tendon of the biceps. Some portion is elevated much above the tendon—the centre presents a slight transverse fissure, which, however, is unattended with much inconvenience. She can draw up the hand towards the shoulder, but cannot extend the arm beyond a right angle.

Eight days after her admission the operation was performed. An elliptical incision included the cicatrix, which was then removed by dissection from the parts beneath. Two arteries were tied. The edges of the wound were brought *transversely* together by adhesive straps, light dressing, and a bandage. A splint was applied to maintain extension.

On the following day there was pyrexia, and on the next an appearance of erysipelas was visible on the posterior surface of the arm. The splint was removed, cold lotion applied, and febrifuge medicine resorted to. The erysipelas attained no degree of severity, and had passed away on the 31st. On this day a splint was re-applied, and a screw was so adjusted as to regulate the extension from day to day.

On the 4th December the wound was dressed with dry lint and strapping. The ulceration healed, the arm became much straighter, and we understand that the operation has proved very successful.

### SECONDARY HÆMORRHAGE.

CASE. *Necrosis of the Thigh—Secondary Hæmorrhage—Ligature of the Femoral Artery—Ultimate Amputation and Cure.**

Jeremiah Chandler, æt. 21, admitted Nov. 21, 1832.

Necrosis of the left thigh—exposed and apparently insulated dead bone felt on the inside just above the knee—much new bone on the outside. Disease commenced three years and a half ago.

On Dec. 13, an operation was performed for the removal of the dead bone. An incision was made on the inside over the new bone, and a small piece was removed by the trephine. The mere application of this was insufficient, and it was necessary to employ the keyhole saw and the trephine again, before the sequestrum could be abstracted. This was nearly four inches in length and one and a half in breadth. No hæmorrhage occurred, nor did any vessel require to be secured.

On the 18th the wound was dressed, and some lint, which had been introduced into its cavity for the purpose of keeping its edges asunder, was removed. The wound had a tolerably healthy appearance.

On the following morning, whilst making some exertion, hæmorrhage suddenly took place, and in less than three minutes three pints of arterial blood had been lost. During the attempt of the house-surgeon to arrest the bleeding another pint had been discharged. It was believed that the hæmorrhage proceeded from the trunk of the popliteal artery and a tourniquet was placed upon it. In the course of three quarters of an hour, the femoral artery was tied by Mr. Hawkins in the upper part of the middle third of the thigh. The patient was feeble, with a languid circulation.

On the 23d there was some fever, with intense head-ache. The wound near the knee was suppurating freely—that in the thigh was partially united, but displayed rather foul suppuration in its centre.

*Haust. salin.*

The fever, &c. subsided in a day or two.

On the 29th, (10th day,) the ligature came away in the dressings.

Jan. 10th. Wound over the artery almost healed—that over the knee is healthy. A small portion of bone has been to-day extracted.

26th. Small fragment of bone extracted again.

Feb. 5th. Another fragment discharged. Strapping applied to-day.

March 2d. Sickness—constitutional disturbance—pyrexia.

*Hyd. sub.* c̄ *Pulv. ant. stat.*

*H. sal.* c̄ *Amm. carb.* gr. v. *Sp. æth. nit.* ℥ss. 6*tis horis.*

4th. Sickness rather diminished—bowels more out of order—wound foul, and a swelling in the groin with little pain.

*Ordered calomel with antim. and opium.*

On the next day the wound had assumed a sloughy aspect, and redness extended in all directions from its edges. He was ordered calomel and opium

* This case having been published, we believe, in the Medical Gazette, we shall greatly abridge the details.

thrice daily, and a nitric acid lotion. On the 7th the wound was still extending. He was put upon wine, with the volatile alkali. This plan was continued, along with the occasional administration of aperients, of aromatics, and of opiates, until the 25th. We have not space for diurnal reports nor particular prescriptions. Their wearisome monotony would fatigue and disgust the intelligent reader.

Long as was the interval between the commencement of the sloughing and the day just mentioned, the improvement in the wound was not very considerable. On the morning of that day it was dressed in the usual manner, and some slight hæmorrhage appeared to have occurred. Soon afterwards, blood of an arterial character issued from the wound in considerable quantity. A tourniquet was applied in the direction of the artery and the bleeding was controlled. Mr. Keate who, by chance, was then in the hospital, laid open a sinus which extended upwards, and made an incision four inches in length. In doing this he seemed to arrive at the sheath of the artery distended with blood, and found that he could command the bleeding by his fingers after the tourniquet was loosened. In consultation it was determined to amputate, and accordingly the circular operation was performed.

The limb was examined immediately afterwards, but the source of the hæmorrhage was not accurately ascertained. No arterial branch was found to communicate directly with the wound, but there was an opening by sloughing into the vein. Several spiculæ of bone were found. The knee-joint was perfectly free from disease.

During the operation he required wine, and after its performance the nicest attention was necessary to apportion the degree of support, and the time at which it should be given or withheld.

On the 28th the dressings were removed and the wound was found doing better than was expected. A small part had united by the first intention, the remainder was granulating, with extensive suppuration. The wound did not advance in a progressive manner, for suppuration became too great, some blood was effused, granulations were absorbed, and part of the union gave way. By great attention to the part and to the health these unpleasant consequences were diminished and removed. A sinus which formed at the lower portion of the stump filled up, and on the 29th April the patient was able to dress himself and lie on the outside of his bed.

Here the report in the ward-book terminates, but we believe that no further untoward occurrence took place.

### Cases of Tumor or Enlargement of Structure in which Iodine was used.

Since this powerful remedy was first introduced to public notice, the opinions of surgeons and physicians have been greatly divided on its real merits. From some it has received extraordinary commendation and a very extensive employment, by others it has been rejected as more dangerous than useful. The following cases may perhaps appear to favour the opinions expressed by the latter. But candour compels us to acknowledge that they prove very little by themselves, and can only be received as trifling additions to the actual collection of facts.

*Case* 1. James Bowles, æt. 33, a smith, admitted November 21st, 1832.

Enlargement of the left femur from about its middle to the knee—tumor formed by the whole bone, which is rough and irregular upon its surface, and apparently not entirely osseous—some pain on pressure, especially on the inner side near the knee—some wasting of the muscles—out of health.

The swelling commenced about the knee five years ago, but was not accompanied with pain for six months. He was then received into the Westminster Hospital and treated by Mr. Guthrie by cupping, blisters, and leeches. From these he received no relief. He now had occasional pain in the hip, worse when quiet than when walking, always aggravated at night, and sometimes extending to the foot. He left the hospital, and subsequently he has followed his occupation at intervals.

*An opiate given each night.*

Nov. 26th. *Ung. hyd. mitior.* ℥j. *Potass. hydriod.* ℈j. *Opii,* gr. x. *M. ft. ung. infric.* ʒj. *femori o. n.*

Dec. 13th. Complains of sore throat, with pain on pressure externally.

The ointment was omitted next day, but on the 16th the pain in the throat was increased, with difficulty of swallowing and pyrexia. By purgation and salines this accidental affection was removed.

Dec. 18th. *Infr. ung.* ʒj. *o. n. iterum.*

22d. *Haust. cinch. ter die. Omr. haust. opiat.*

He continued on this plan with little material alteration till the 16th of January, when he was made an out-patient. At this time the pain and the tenderness were gone, and he thought that on the whole the thigh was better than it had been from the first. The enlargement, however, remained undiminished.

As an out-patient he employed the following ointment.

*Ung. hyd. mitior,* ℥j. *Potass. hydriod.* ℥ss. M.

On the 10th April the following report was entered on the book.

Swelling of the lower part of the bone near the joint removed—the central portion continues in nearly the same condition. No pain now, but he has had some since his dismissal from the house. The health is not good.

*Case* 2. Charles Wolger, æt. 23, a bricklayer, admitted Nov. 21, 1832.

Fulness over the front of the left femur, near the trochanter—a sensation of crackling, probably seated over the bone, experienced on moving the femur quickly—no pain on pressing the head of the bone into the socket—wasting of the muscles of the thigh from a little above the middle to the knee—half an inch of shortening of the limb.

Two years ago he first felt pain in the knee; it usually went away when he was quiet. One year ago the hip became painful, and gave him the idea of something being broken in the joint. He has followed his occupation till five weeks ago. He has been cupped upon the hip, and the knee has been repeatedly rubbed without benefit. The health has been and still is good.

27th. *Ung. hyd. mitior,* ℥j. *Potass. hydriod.* ʒss. M. *Inf.* ʒj. *o. n. femori.*

The enlargement seemed at first to decrease in size, and this, with perhaps a more close examination, enabled the following facts to be distinguished.

There appeared to be a tumor in front of the femur, opposite to the trochanter, and a little below it, leaving the neck of the bone quite free. The tumor was about three inches in length and two and a half in breadth, apparently firm, but not having exactly the feeling of bone, and probably covered in part by fluid. It seemed to be attached to the bone, and was covered by the vasti and the rectus.

From Dec. 27th to Jan. 5th, three blisters were applied to the groin, or behind the trochanter. The hydriodated ointment was continued.

16th. The tumor, which had diminished to half its former size, has lately increased, and is larger than it was before his admission. It projects a little more towards the outside of the femur, and occasions no obstruction to the motion of the hip-joint.

On puncturing the tumor with a needle a little serous fluid seems to issue. The sensation communicated by the passage of the instrument is as though it passed through cartilage, and finally struck against bone, more anteriorly than the natural outline of the femur.

The health beginning to suffer in some degree, tonics with acid were exhibited. On the 26th of February the tumor had increased at the back of the trochanter, and was felt extending upwards under the glutæi, which were wasted over it. The softer portion of the tumor seemed in some measure susceptible of the influence of the ointment, but the harder structure certainly resisted it.

Soon after this report the patient became affected with cough and such general ill-health, that his case was confided to the care of the physician. Previously to this, a caustic issue had been inserted behind the trochanter, but had not been productive of the slightest advantage.

On the 8th of May the patient was dismissed, the iodine, of course, having failed in his instance.

### Extensive Idiopathic Phlebitis cured.

*Case.* Jane Day, æt. 24, admitted Dec. 19th, 1832.

A chain of abscesses displayed in the course of the saphena major and saphena minor. Leg and foot much swollen, and a large abscess extending from the ham to the ankle—another smaller one on the outside of the leg.

A month ago, while walking up stairs, was suddenly seized with violent pain in the knee, succeeded by pyrexia. In the course of 24 hours, the leg and thigh presented a swelling, commencing at the ankle, and extending up the inside of the thigh, in the course of the saphena vein.

Such were the symptoms and such the history of this complaint. Mr. Hawkins opened both abscesses of the leg, discharging from the greater about twelve ounces of matter, and from the lesser about two or three. The patient was ordered aperients, opiates, and, after the febrile disturbance had subsided, cinchona.

After the evacuation of the matter from the abscesses, the swelling of the leg diminished. There were now two openings on the inside of the thigh, just above the knee, which communicated with the veins, and which gave exit to pus, in considerable quantity, from the veins themselves. Pus could also be distinguished in different parts of the veins, as high as the groin.

She was put upon fish diet, and on the 24th she was ordered wine. On the 27th, a small abscess was opened in the upper part of the thigh.

In a day or two, the leg was in a state to bear pressure by bandages and strapping. Two small sinuses in the thigh were laid open. The abscesses filled up and healed by degrees, the improvement being perceptible first in the lower. The skin and the cellular membrane recovered their natural appearance. The general health was improved pari passu, and the patient was cured about the middle or latter end of the month.

### Wound of the Orbit by a Pitchfork.

*Case.* John Bramwell, æt. 35, admitted Jan. 9th, 1833.

The prong of a pitchfork had been thrust into the left orbit, between its floor and the ball of the eye. Immediately on the receipt of the injury he was sick. He went to an apothecary, who gave him some laudanum, and advised him to apply directly at the hospital.

The direction of the external wound was transverse, and its length was about an inch. The edges were not contused. The cornea was flaccid, and had been ruptured at its inner part, the rupture appearing to have extended into the sclerotica, through which the choroid, iris, and part of the vitreous humour protruded. The whole of the organ was suffused with blood, apparently from the wounded choroid.

He complained of much pain in the eye—the pulse was weak and feeble, and the skin was cold. He was placed in bed, and cold lotion was applied. He vomited soon afterwards.

At 5, p.m. three hours after the accident, he had vomited some blood, supposed to proceed from the frontal sinus or æthmoidal cells. The pulse being strong, venæsection was ordered, but was prevented by another attack of vomiting, which occasioned some subsequent collapse.

6, p. m. *V. S. ad* ℥xviij.

*H. salin.* c̄ *Vin. a. tart.* ʒss. 6*tis horis.*

10th. Had pain in the eye last night. He vomited a small quantity of blood. There are no unpleasant symptoms today. Bowels not opened since his admission.

*H. sennæ q. s.*

Nothing worthy of notice occurred till the evening of the 12th, when the patient complained of pain in the head and of throbbing, intolerance of light, and tendency to sickness. The bowels were confined.

*V. S. ad* ℥xij. *H. sennæ.*

The blood was much buffed and cupped. On the 13th the pain in the head had abated, and the pulse was 60 and soft. The bowels were still disposed to be confined. Their condition was, of course, attended to.

On the 19th, more pain in the eye and in the forehead.

*Hirud.* vj. *tempori.*

21st. Cornea clear—iris drawn inwards—blood effused into the upper parts of the eye—protruded parts slightly ulcerated. The wound in the integuments is closed.

*Protrusion touched with arg. nit.*

22d. More pain in the eye.

*Hirud.* viij. *tempori.*

The patient was now put slightly under the influence of calomel.

Feb. 2d. Some pain in the eye—less vascularity of the conjunctiva. The iris is clear, but the pupil is distorted and drawn towards the inner canthus. A small ulceration where the original wound was situated.

On the 6th he was made an out-patient, and a lotion of sulphate of zinc was prescribed. In the course of a short time he discontinued his attendance, and the ultimate condition of the eye is unknown.

Here the present report must conclude. The cases are possessed of practical interest, and derive, as we remarked, an increased importance from the circumstance, that, taken under the immediate inspection of Mr. Hawkins, the acknowledged zeal and attention of that gentleman are a guarantee for their strict fidelity. We trust that on future occasions we may have it in our power to present a more complete and satisfactory account of the facts that occur in Mr. Hawkins' practice, of the treatment he employs, and the views that he adopts.

---

### IV. HÔPITAL DE LA CHARITÉ.

[*Clinique of M. Bouillaud.*]

### RHEUMATISM.

Active and repeated depletions, both local and general, are invariably employed by this physician, against all inflammatory articular affections. There was a man, lately in the wards of this hospital, who was not cured, till after the tenth bleeding; and at the same time, there was a second case of a patient who had been under treatment at another hospital for several months without any benefit—two large bleedings in the course of one day (the disease having assumed an acute character) almost at once brought him into a state of convalescence.

[Our readers may judge for themselves of what the reporter of the La Pitié Hospital sneeringly denominates "le traitement de la Charité."—*Ed.*]

### CHRONIC ICTERUS.

Three out of seven cases recently admitted into the wards, died. In two of these cases, the ductus communis choledochus, was found completely closed by biliary calculi; and in the remaining one, the liver presented extensive tuberculous deposit.

### TYPHUS FEVER.

A young man aged 19, of a sound constitution, residing in Paris for the two preceding months, was admitted under the care of M. Bouillaud on the 29th June.

For five days he had been ailing with headache, general feebleness, and repeated shiverings. He was bled from the arm; and on the following day. M. B. visited him. There was considerable prostration; his answers were slow, and confused; his breath very offensive, tongue dry; he had much thirst and a disagreeable bitter taste in his mouth. Pulse 70, of moderate strength; respiration natural; abdomen soft and not tender on pressure. No information as to the state of the bowels could be obtained; anorexia, but no nausea; intense headache.

*Forty leeches to the abdomen; and soothing demulcents ordered.*

July 1st. No amendment.

*Thirty leeches to the abdomen.*

2nd. Less headache; diarrhœa; great prostration; pulse from 56 to 64. Stools pass from him unconsciously.

*Thirty leeches behind the ears; cold affusion to the head.*

3rd. During the employment of the cold affusion, high excitement, succeeded by coma, came on. This stupor continued until death; a petechial eruption on the abdomen and thighs.

The same treatment ordered to be persisted in: also a few grains of tartar emetic to be mixed in a ptisan; and a dose to be given occasionally.

5th. He died.

*Dissection.* Abdominal viscera healthy —intestinal follicles not affected. Well marked appearances of minute injection, and of congestion of the encephaloid—the medullary substance when cut, exhibiting an infinity of red points from which the blood oozed out freely in large drops; the pia mater was so highly injected in some places, that it seemed as if blood was actually effused. Considerable quantity of serum in the ventricles.

The reporter (M. Pelletan) of the preceding case admits that the cephalic character, or type of the disease, was not sufficiently attended to, at its commencement; the physician having supposed that the primary seat of the mischief was in the intestines; which however on dissection proved to be quite sound.

An apt example of the Sangrado practice of the Broussaists. Our waggish friend at the La Pitié should call it " le traitment de la non-Pitié, rather than " de La Charité;" for certainly there is a want of pity, and no charity in such insatiable blood drawing.—*Ed.*]

### Internal Use of Croton Oil.

Various expedients have been employed to counteract the irritating qualities of croton oil, and a multitude of formulæ recommended for its preparation; but we have generally found the simplest method, the best; viz. that of giving a drop or two in a spoonful of ptisan. The pills of M. Caventon, we have also tried extensively, and we on the whole approve of them; they are prepared thus. Take of croton oil, five drachms; of soap-lees, two drachms and a half; mix and rub them well together in a glass mortar, until a soap is formed, which is then to be made into moulds: But after all, neither these pills, nor any of the other formulæ insure so speedy and brisk an operation as the same quantity of the oil swallowed with a spoonful of liquid. It is well known that it generally produces a feeling of heat in the throat, extending to the pit of the stomach; also nausea, and sometimes vomiting; but this effect is more frequent in women than in men. It has been given repeatedly in cases of gastro-intestinal inflammation, and seldom has it caused any accidents. The diseases in which its internal use has been especially beneficial, are painter's colic, and severe cephalalgia; the speedy relief it often brings to the former, is gratifying alike to the patient and to the physician. One of the patients who was treated with it, had suffered repeated attacks before, and had been subjected to what is called " the La Charité treatment," [We presume this alludes to the leeching, and leeching, and leeching again, recommended by some of the disciples of Broussais.—*Editor.*] against a repetition of which he now vehemently protested. A dose or two of croton oil cured him. The fumes of copper will sometimes occasion all the symptoms of genuine colica pictonum:—a similar treatment should be pursued. We deem it unnecessary to report any of the cases, which exhibit the utility of croton oil against obstinate headaches. It is indeed an admirable remedy, and often works like a charm.

### Acute Rheumatism, Pleuro-Pneumonia and Pericarditis.

A young man, aged 18, admitted under the care of M. Dalmas on the 28th June, 1833. He is of a delicate and lymphatic constitution, and, of late, has been obliged to sleep in a damp chamber. Three days ago, he was suddenly seized with severe rheumatic pains in his joints, more especially in the knees, and with general pyrexia. These symptoms have ever since been on the increase. The knees are now swollen, inflamed, and cannot be moved without causing great pain; pulse full, strong, and frequent.

*Prescript.—Mel. boraginis. Pulv. Doveri*, gr. iij.

*V. sectio, ad* pat. iij.

29th. Same as yesterday.

*Rep. V. sectio.*

30th. The pains are now fixed more in the upper extremities, and especially in the shoulders.

*Mel. borag. P. Dov.* gr. viij.

*V. sectio.*

July 2d. A sudden change this morning. The articular pains have left him entirely, and there have supervened great oppression, hurried and painful breathing, sharp pain at the lower part of both sides of the chest, increased by the efforts of a dry and teasing cough, and most severe at the left bosom. Auscultatory symptoms of pleuro-pneumonia on each side.

*V. sectio. Mel. borag. Hirud.* xv. *mammæ sinistræ.*

4th. Percussion sonorous over the upper and back part of the chest; at and below the level of the spine of the scapula distinct œgophony, absence of the respiratory murmur, and dulness on percussion. The pneumonic symptoms have abated.

*A blister below the left mamma—diuretic medicines.*

9th. The pleuritic effusion on both sides has sensibly diminished. Bled to two cupfuls.

11th. The use of gentle purgatives (ol. ricini) commenced.

13th. Twenty leeches over the heart.

17th. Bruit de soufflet heard over the region of the heart. Ptisan of digitalis—twenty leeches on the præcordial region.

19th. Sinapisms to the calves—purging mixture—blister over the heart. Malaga wine allowed.

20th. Bruit de soufflet no longer heard. An evident outward fulness of the cardiac region. Dulness on percussion over a great extent.

26th. Bruit de soufflet again heard to-day, for the first time since the last report; the præcordial fulness has abated; the extent of the dulness diminished.

28th. No longer any apparent oppression; a dull, deep-seated pain felt at the cardiac region—it is increased by lying on the right side. When the chest is dilated by inspiration, it is observed that there is a vaulted fulness over the heart, and the intercostal spaces seem somewhat widened, and are rather more prominent than at other parts. The contour of the left side of the chest, measured under the mamma, is half an inch greater than that of the right side. A well-defined and well-marked dulness, on percussion, extends over all the vaulted portion. The pulsations of the heart are frequent and strong, but not loud; they can, however, be heard distinctly on the right side. A very distinct bruit de soufflet over the heart and the carotid arteries. No purring tremor felt. Pulse 102, strong and hard. Percussion and auscultation announce that there is still a pleuritic effusion. Respirations 28 in the minute. The patient went on improving until the—

31st. When, in consequence of some imprudence in diet, the pulse rose to 114, beating full and strong, the breathing was oppressed and quick, and the general expression of the patient much worse.

Aug. 4th. Respiration easy, and numbering only 20 in the minute—pulse 96, more soft and calm—bruit de soufflet less distinct—dulness on percussion not so extended, and the arching or prominence of the cardiac region has fallen considerably. Upon measuring the two sides of the chest, the left is found to be only three lines larger than the right. The decubitus is much more easy than hitherto.

7th. The symptoms have been gradually on the decrease since last report. The bruit over the heart and arteries is now very feeble; the pulsations of the heart, although much more tranquil, are still rather dull, and, as it were, very deep-seated; and if the ear be applied for some time with great attention, a sort of gurgling noise, like that of the waving of water, may be heard;

—a phenomenon probably owing to the diminution of the serous effusion.

12th. Discharged well—only weak.

*Reflections.*—The preceding case illustrates well these particulars. An erratic, acute rheumatism, of eight days' standing, suddenly recedes, and immediately all the symptoms of a double pleuro-pneumonia set in. In the progress of the case, without any sufficiently-appreciable cause, pericarditis supervenes; the inflammatory symptoms are quickly followed by those of effusion into the pericardium—there is pain, great extent of dulness on being percussed, outward fulness or prominence, loud bruit de soufflet over the heart and the carotid arteries, tumultuous action of the heart, extreme anxiety, and even delirium. On the 4th day, the effusion was at its acmé, and we find that the bruit de soufflet had disappeared. On the ninth day, the symptoms decrease in severity; absorption had probably commenced; the bruit de soufflet could be heard again; and from this date the patient went on progressively to a complete recovery.

---

V.

### THE PRECEPTOR.

### Clinical Lectures.

Dr. Stokes.—In this department of our review, we should do injustice to a meritorious member of the profession, if we omitted to notice the clinical lectures of Dr. William Stokes, delivered at the Meath Hospital, Dublin, and published by our active and zealous contemporary—the London Medical and Surgical Journal. Although clinical lectures are more susceptible of analysis than those that are merely elementary, yet we shall notice only the more prominent facts and features of these bed-side observations.

1. *Phthisis.* Dr. Stokes makes some useful remarks on the hectic fever which so often attends phthisis, even before the tubercles break down, and matter is discharged from the mouth. Extensive tuberculation in the lungs will, in fact, occasion hectic fever in many cases; while a vomica, the greater portion of lungs being sound, will present no hectic at all. Dr. S. has been giving a trial to the chlorine inhalations, so eulogized by M. Cotteran in Paris; but, as might be easily predicted, none of those cures of actual phthisis, as vaunted by our Parisian cotemporary, have been witnessed in the Meath Hospital. In some cases, it *appeared* to do some good—in far more it did positive mischief. All those remedies which check the expectoration, do more or less harm, by increasing the inflammatory action in the surrounding tissues of the lungs. In a case of gangrenous abscess in the lungs, great benefit was obtained from the chlorine inhalation.

2. *Pleuritis Biliosa.* Dr. Stokes alludes to a case in the hospital, where effusion took place in the right pleural cavity after inflammation. The side was bulged out and the intercostal spaces enlarged, with absence of respiratory murmur, resonance of the voice, and vibration when speaking, as felt by the hand on the opposite side. The patient was in a low grade of health, and the complaint was complicated with gastric symptoms, so as to present what the older authors would have termed bilious pleurisy. Leeches to the epigastrium and to the affected side, were producing beneficial effects.

We perceive that Dr. Stokes uses iodine in dropsy. He cautions his pupils against neglect of the bowels in dropsical patients, however weak or depressed they may be, since fæcal accumulations always increase the anasarcous swellings.

3. *Dysphagia.* An interesting case of dysphagia is related in these lectures, Dr. Stokes having first enumerated the various causes that produce this distressing state. The case is, abstractedly, this:—Edward Lynch was attacked ten days before admission, with pain in the back, and stitches at the lower part of the sternum. These continued for a week, when he first felt a soreness on swallowing, which increased so much, that on the following day he could swallow no solids whatever, the attempt producing great pain, and a sense of weight, followed by hiccup and vomiting. He has frequent eructations with relief of the sense of oppression. Since the occurrence of the dysphagia he has lost his appetite; there is some epigastric tenderness; bowels costive since the commencement of his illness; his general appearance is that of a man labouring under low gastric fever. On examination, by the stethoscope and percussion, no morbid phenomenon could be detected, *except that the respiration over the whole of the right lung was feeble as compared with that in the left, no sound continuing clear on percussion.* On the day after admission, Mr. Porter passed a probang without meeting any obstruction, but the patient complained of soreness at the seat of stoppage.

Before the probang had been used we considered that the irritation was very low down, from the statements made by the patient himself. This morning he says he feels better, but as yet he has not attempted to swallow fluids. We have observed, that some minutes after he has swallowed a morsel of food, symptoms of irritation of the stomach come on, he begins to eructate, and goes on in this way until he gets up a small quantity of frothy fluid."

Dr. Stokes enters again on the various causes of dysphagia, examining them seriatim, and trying whether or not they will bear on the above case. He comes at length to the conclusion, or rather conjecture, that the cause is either acute œsophagitis, or disease of the cardiac orifice of the stomach, or both. We were inclined to agree with him; for the sudden advent of the complaint, and the no less sudden amelioration, while in hospital, seemed to negative the idea of there being any tumor or other mechanical obstruction to the deglutition in this case. The treatment was leechings, iced water, blisters, and purgative injections. In a subsequent lecture, the true state of things was revealed by the knife, for the patient died. Immediately before this event, the patient vomited a large quantity of blood. On examination the cause of the dysphagia was found to be a small aortic aneurism, the size of a walnut, which had perforated the œsophagus, and pressed on the opposite side of the tube, where the lining membrane was inflamed and extensively ulcerated. The cardiac portion of the stomach was inflamed, and the aorta shewed marks of extensive recent disease. The case is highly interesting.

4. *Diptherite.* This was a man admitted on the 10th of August, labouring under double pneumonia—intense and neglected inflammation of both lungs. He was treated by venesection, leeches, and antimony. He was decidedly relieved of the pneumonia. He took eighteen grains of emetic tartar in three or four days, and bore it well. On the fifth day, however, a new train of symptoms appeared. His cough assumed a laryngeal character, with difficult articulation, laborious breathing, and soreness in the throat. On examination, several thick patches of dense white substance were seen on the tongue, velum, and back of the pharynx. The antimony was discontinued—a large blister was applied to the throat—while the exudations were freely brushed with the strongest muriatic acid. In two days he was improving rapidly, the patches being nearly detached. Some relapse, however, occurred, and the same remedies were again administered, a brisk purgative being given. Recovery then took place.

Dr. S. has made some practical remarks on this case. He observes that the disease is a dangerous one—because, if the inflammation extends to the larynx, the patient may die with all the symptoms of croup. He strongly recommends the muriatic acid, as originally prescribed by M. Bretonneau.

5. *Laryngeal Phthisis.* We were much pleased with Dr. Stokes' observations on this case; for we quite agree with him, that it is very rare to find ulceration of the larynx unaccompanied by disease of the lungs—and moreover we have seen several cases which were pronounced by able practitioners to be laryngeal disease or phthisis, which proved, on dissection, to be phthisis pulmonalis, without any laryngeal disease at all. We should not, therefore, except with great caution, exhibit mercury in such cases—and still more caution should be used before we have recourse to tracheotomy.

"I remember (says Dr. S.) having witnessed a case in which an error of this kind was committed; the patient was a gentleman, labouring under an inflammation of the mucous membrane of the larynx of considerable standing, which owing to some cause was much exacerbated; he had been mercurialized for it, and when I saw him, he was like a person in the last stage of consumption. He had great rapidity of pulse, emaciation, hectic, and profuse expectoration. On applying the stethoscope, in order to satisfy myself, I found several large caverns in the substance of the lungs, which must have existed there for a considerable time."

Dr. Stokes remarks, that the stridulous breathing, in laryngeal disease is unfavourable to the exercise of auscultation. But it does not effect percussion. If, therefore, we meet with a patient who has been labouring for some time under laryngitis—who has acceleration of pulse and wasting of flesh—and who, on examination, presents dulness of sound in the upper portions of the lungs, we may be almost certain that tubercles exist there. In most cases we shall find that pulmonary symptoms preceded the laryngeal affection.

6. *Dropsy*. Dr. Stokes alludes to a case in the hospital where dropsy succeeded to several inflammatory attacks in a young woman. After bleeding and other necessary evacuations, the physician was using iodine, both internally and externally. The internal form is what Dr. S. denominates the "*iodine mineral water*," composed of one grain of pure iodine, and eight grains of the hydriodate of potash to two pounds of distilled water—which quantity is to be taken in 24 hours. Under this plan the abdomen was reducing in size, and the patient amending. Dr. S. strongly recommends iodine in dropsical affections. We believe we were among the first to introduce this medicine to the notice of the profession, in consequence of a remarkable case that was treated in this manner by Dr. Gordon, Dr. Johnson, and Mr. Guthrie, with complete and permanent *success*.

### VI. Sympathy.

Dr. Stokes, in one of his clinical lectures, now publishing in our contemporary, the Medical and Surgical Journal, makes the following remarks on the subject of sympathy, which are, generally speaking, very judicious, though not entirely free from animadversion.

"We come now, gentlemen, to the consideration of the laws of sympathy. When we consider the system in a state of health, we find that the effects of impressions made upon one organ are rapidly transmitted to others. We observe, too, that excitement of one organ produces excitement in another and a distant one. These are the sympathies of health; and the organs of their transmission are supposed to be the nerves. Now, in disease we see the same phenomena; and Broussais maintains, that the morbid sympathies only differ from the healthy in this—that they transmit more irritation, or, to use his own words, a mode of excitation repugnant to the vital laws. It is plain, that if by the latter expression he means anything more than a plus degree of the natural excitement, he is departing from the principles of his doctrine.

Broussais divides the sympathies into two classes,—sympathies of relation, and sympathies of organic life. What does he mean by this? Modern physiologists have divided the functions into two classes,—those of animal and those of organic life. The centre of organic life is the great sympathetic nerve; the central organ of animal life is the brain and spinal cord. To give an example of sympathies, let us take a case of inflammation of the mucous membrane of the intestinal tube. During the course of the disease the patient gets headach—

here is a sympathy of relation; next, we observe convulsions—here is another effect of sympathy; he becomes delirious—another similar result of the same cause; and all these are sympathies of relation. Let us go further. The same patient, as the result of an enteritic attack, gets fever, heat of skin, excitement of the circulation, and jaundice—all these are organic sympathies. Again; during the course of the same disease the patient may get an attack of cough and a difficulty of breathing—here is another organic sympathy, for the respiratory organs are affected. When the sympathies of relation are in excess, the results of that excess may vary according as they are reflected on the nervous system; and if these be very violent, the person may die of the excitement of the organ of animal life. If the sympathies of organic life happen to be excessive, the patient may be destroyed by the transmission of disease to other viscera: he may die of disease of the lungs or liver, or some other organ. This may tend, in some degree, to explain the laws of sympathy. On this subject Broussais has announced propositions too numerous to be laid before you in the course of a few lectures. To one of them, however, I would particularly draw your attention, namely, that sympathetic functional derangement when excessive and long continued may ultimately become real, or, in other words, that the affection of an organ, when persistent, may, though at first functional, afterwards become organic. Take the example before given, of a patient labouring under enteritis, with severe headach. If the disease continues for a long time the brain may become inflamed, and the patient die of cerebral disease. Two conditions, therefore, may produce the conversion of sympathetic irritation into real disease, intensity of symptoms and long continuance.

Let us take a few examples of the first class of diseases, or those which arise from an intensity of sympathy. A patient gets an attack of hepatitis, or pneumonia, or bronchitis; the action of the heart is disturbed or excited, the disease continues with undiminished intensity, and the heart, which was originally only affected by sympathy, finally experiences an organic change. Again; a person is seized with severe gastro-enteritis; during the progress of the disease his breathing becomes hurried, he gets cough and other symptoms of a pulmonary affection, and finally, intense and fatal pneumonia. A child labours under irritation of the brain; this produces vomiting, and the vomiting may ultimately terminate in gastritis. On the other hand, we may have hydrocephalus supervening on an intense gastritis. Again; to take instances of disease arising from sympathy, with chronic and long-continued affections, how often do we read of persons, having constant headach from chronic gastritis, finally dying of disease of the brain. In the same way a gastritis may, in course of time, bring on hepatitis, or perhaps disease of the lung, forming what has been termed *dyspeptic phthisis*. How frequently is morbus cordis the result of long-continued pulmonary affections. Does not long-continued painful menstruation frequently end in organic disease of the uterus? These are examples of persistent sympathetic irritation terminating in organic disease, and tending to establish the great rule, that long-continued functional lesion is closely connected with that process which produces organic change. *It is, therefore, unscientific and dangerous to prescribe, on all occasions, for a chronic functional affection, on the supposition that it is only such; it is often safer to consider it as a disease of the organ itself.* Let us see how this is borne out by facts. It is now admitted by the most enlightened pathologists, that in cases of mania which have been going on for years, there is always more or less of arachnitis or of disease of the substance of the brain, and that the patients die with symptoms of a cerebral affection—as convulsions, paralysis or coma. Let a patient labour for a considerable space of time under severe palpitations, and the result may ultimately be disease of the heart; or let him be dyspeptic or asthmatic, and he will commonly get a gastritis or organic disease of the lung."

In the passage which is marked in italics, by our author himself, we conceive that the position is not well stated. We think it ought to run thus:—"*it is dangerous to prescribe, on all occasions, for a chronic functional affection, on the supposition that it is* SYMPATHETIC; *it is often safer to consider it as a disorder or disease of the organ itself.*"

How often have we to lament the mistake which is daily made by practitioners, in respect to dyspeptic phthisis! Cough and other symptoms of pulmonic affection are attributed to disordered digestion, till the disease of the lungs is irremediable! The term "DYSPEPTIC PHTHISIS," annually consigns several thousand victims to an early grave in this country!

---

VII. MEATH HOSPITAL, DUBLIN.

CASES OF INTERNAL ANEURISM.*

These cases are intended to exemplify the symptoms, and the want of symptoms, that characterize aneurism of the commencement of the aorta. They are also brought forward for another purpose, to which we shall allude when we have related the details. As our object is to communicate, as far as a journal can do so, that practical information which is only acquired by the actual study of cases, we will give as complete and concise an account of the prominent features of the present facts as their value may require and our limits will permit.

CASE 1. *Aneurism of the aorta opening into the œsophagus—symptoms chiefly those of dysphagia.*

E. Lynch, æt. 26, a shoemaker, of intemperate habits, admitted into the Meath Hospital, March 19th, 1833.

Has eructations, about every five minutes of a frothy watery fluid, sometimes acrid and sour—difficulty of deglutition, the attempt always producing great pain and oppression at the lower part of the sternum, followed by hiccup and by vomiting—loss of appetite—tenderness over the epigastrium—and constipation. Being desired to swallow a morsel of bread, he did so, but said it stopped in the passage; after repeated draughts of whey it passed down, but not without a good deal of spasm, resembling hiccup. It was not vomited.

On examination, the chest sounded well on percussion every where, and the stethoscope discovered no sign of disease in the heart or aorta. The action of the heart was a little stronger than natural, but the sounds were healthy. Respiration *feeble*, but pure in the upper part of the right lung; pulse about 90.

The symptoms had commenced ten day previously with pain in the back, stitches in the chest, and constipation. The difficulty of deglutition had appeared only two days before his admission.

On the 21st, Mr. Porter, at the request of Dr. Stokes, introduced a probang into the œsophagus, without encountering any decided obstruction. He was sensible, however, that the instrument passed over a soft tumour. The case seems to have been treated as one of gastritis, and leeches to the epigastrium, ice, and an anodyne procured a temporary and slight relief. As our object is mainly to display the symptoms and the course of these cases, we may pass with a cursory notice the items of an ineffectual treatment.

On the 23d, the patient complained of pain in the spine of the right scapula, occurring seldom, and generally at the end of a deep inspiration. It imparted the sensation of the scapula being lifted up. This particular symptom was mitigated by a blister. On the 26th he made an effort to get down some tea; this produced great distress, and was followed by hiccup, retching, and vomiting of a large portion of it.

On the 27th he complained of weight in the centre of the chest, and has frequent suffocating cough. In an attempt to swallow, he feels as if something stopped the passage, and prevented any thing passing up or down; this stops his breathing also, and subsides suddenly.

* Dublin Journal, Nov. 1833.

Whilst speaking, a severe fit of retching came on, which continued several minutes, and he threw up some mucus slightly tinged red. He then swallowed a few sups of drink, but immediately rejected about an ounce with the same coughing and eructation.

At 4, p. m., he had an attack of cough and of vomiting; he threw up a pint of florid blood, and immediately expired.

*Dissection.* A large coagulum filled the stomach. On slitting up the œsophagus, a clot, much larger than a pigeon's egg, and covered only by the mucous membrane, was seen projecting into it. Its situation was nearly three inches from the cardiac extremity. The mucous membrane had given way by ulceration in one spot, and thus was furnished the blood that filled the stomach and that which had been vomited.

" On opening the aorta, the pathology of aneurism, as connected with acute arteritis, was beautifully illustrated. The lining membrane was of a bright crimson or carmine colour, varied with small spangle-like patches of a paler and more opaque tint. This vascularity resided principally in the lining membrane, for on stripping off a portion of it, the fibrous tissue, although evidently inflamed, was much paler. The patches above-mentioned were caused by depositions of a *soft, white, cheesy substance,* which were either in the lining membrane or between it and the fibrous coat; it came off attached to the lining membrane.

There were three aneurisms in different stages of progression; one, the largest, communicated with the clot, which had burst into the œsophagus; the opening into the aorta would admit the point of the little finger. Another, within about half an inch of the former, was about the size of a hazel-nut, its opening into the aorta being about the diameter of a crow-quill; its internal surface was smooth, as if lined by the inner coat of the vessel; the middle coat terminated abruptly by a thick cellular edge at the opening, and its external covering seemed to be formed of the cellular coat together with the pleura. The third was only in its commencement; a slight deviation from the level of the lining membrane was seen in the centre of one of these opaque spots, under which the fibrous coat was thinned and beginning to be absorbed.

The large tumour had made pressure through the œsophagus on the right bronchus at its posterior part, and thus caused the feebleness of respiration observed during life in the right lung.

The lungs were healthy: the heart paler and softer than natural."

It is to be regretted that the scarlet appearance of the aorta is not described with more accurate minuteness. The distinction between the colouration of staining and that produced by actual arteritis, is so doubtful and so difficult, that the practised pathologist hesitates to admit a statement unsupported by convincing evidence.

CASE 2. *True Aneurism of the Ascending and Transverse Arch of the Aorta, bursting into the Pericardium—Symptoms of Bronchitis.*

Pat. Walsh, æt. 26, a carman, of intemperate habits, admitted into the Meath Hospital, July 23, 1832.

" Face puffy and tumid; neck much swollen, apparently from serous infiltration; the jugular veins turgid and tortuous; there is no œdema of the extremities; he has frequent, short, bronchitic cough, with frothy expectoration, and complains of stinging pain in the right shoulder, shooting down the breast, and frequently catching his breath; the cough and dyspnœa are always worse at night. Sleep bad, broken, and accompanied by frightful dreams. Appetite very good; some thirst; tongue clean and moist; bowels regular; pulse 100 and equal, perhaps a little more feeble in the right arm than in the left; slight pain on pressing the larynx, and some difficulty of swallowing, which he attributes to the tumefaction of the neck.

*Stethoscopic phenomena.*—Impulse of the heart heard very loudly over the entire chest, but no ' bruit de soufflet ' discoverable in any part of it. The anterior portion of the chest sounds dull generally, but particularly in the right subclavian region, where there is feeble respiration, and some sonorous rale. In the right acromial region there is distant mucous rale, with tracheal respiration; over the whole right side respiration is feeble; it is puerile over the left lung; posteriorly the chest sounds clearer. On laying the hand upon the chest, no increase of action in the heart can be perceived."

He states that he has had an habitual short cough for the last four years, but that within the last ten days it has become greatly aggravated. He has also been subject to dyspnœa on exertion, and palpitations for the same space of time; he never spat blood, nor knows he any cause for his ailment, unless a fall on his right shoulder some years ago.

The general symptoms were obviously those of disease of the heart or the aorta, attended, as it commonly is observed to be, with bronchial inflammation. The diffused cardiac impulse and the loud sound (for Mr. Porter's language is not critically accurate) detected by the stethoscope must confirm the suspicion which the symptoms would create. The feeble respiration in the right side of the chest, the sonorous rale, and the dulness on percussion might indicate, if taken by themselves, imperfect consolidation of the lung from inflammation, or compression exercised by some unnatural growth. The latter idea would be countenanced and strengthened by the general symptoms of aneurism, and the stethoscopic examination of the heart. We do not prefer these remarks with the paltry affectation of superior acuteness, but in order to display the process of reflection that would occupy the mind of the well informed physician and the rational stethoscopist.

The patient remained in the hospital five days, and was benefited by the judicious means that were employed. His symptoms soon returned, and he entered another Institution. After remaining within it for a fortnight, he suddenly dropped dead in a moment of exultation.

*Dissection.*—" On opening the thorax, a large tumour presented itself, extending from the diaphragm to the level of the lower border of the first rib on the right, and stretching across the upper bone of the sternum to its left side, where it terminated. The vena innominata crossed the front of it, and must have suffered compression, so closely did the tumour approach the sternum. The tumour was evidently formed of the pericardium, into which the aneurism had burst by a small valvular opening, of size just sufficient to admit a quill, and thus the bag became distended with coagulated blood and serum. The aneurism *seemed to be a true one,* and to be formed of the aorta dilated as far as the origin of the left subclavian artery; the dilatation commencing at the very root of the vessel, where it measured three and a half inches in diameter, and gradually diminishing to its termination. The lining membrane could be traced throughout, and under it was much soft ætheromatous substance. The coats of the artery were in many places become extremely thin, and the right lung greatly compressed, especially at its superior part, adhered throughout to the sac."

Case 3.—*Aneurism of the Aorta, passing between the Œsophagus and Trachea —Symptoms of Laryngitis.*

On the 11th Aug. 1827, Mr. Porter was called to see a drunkard, æt. 24, a nailor, with the view of his performing tracheotomy for acute cynanche laryngea. He was supported upright upon his bed of straw—his arms extended—his hands clenched—his chest heaving convulsively—his face pallid and swollen—his lips transparently blue—his eyes staring, and his entire body covered with profuse perspiration. His respiration was sonorous and laboured, but the trachea was not moved rapidly up and down the neck. Pressure on the larynx externally gave no pain whatever, and on passing the finger into the fauces, the epiglottis could be felt of its natural size and texture. The symptoms had already lasted seven days; and, doubting the nature of the case, Mr. Porter prudently postponed the operation. The patient expired in the course of the day, with every symptom of oppressed brain, produced by protracted strangulation.

*Dissection.* The larynx and trachea seemed perfectly healthy. The lungs did not collapse to the degree which they usually do; their cells were loaded with a reddish frothy mucus.

Between the trachea and œsophagus, just below the root of the neck, a small tumor was observed, which proved to be an aneurism of the arch of the aorta, springing from between the left carotid and subclavian, and seeming to involve the origins of both vessels. Its size was about that of a very large walnut, but with the sternum removed, and in the collapsed condition of the parts, it could not be ascertained, whether the pressure it must have exerted on the trachea was sufficient to cause suffocation and death. However, there was no other mode of explaining it.

The internal coat of the aorta exhibited a bright pink or rose tint, mottled with many specks of a paler hue, which were found to be soft and steatomatous depositions. The *opening* into the sac was smooth, but the sac was not cut into, nor was the structure of its walls examined.

Mr. Porter remarks that, although the aneurism must, apparently, have pressed on the œsophagus, he had never been informed of the existence of dysphagia.

Case 4.—*True Aneurism of the Arch of the Aorta—Symptoms not obscure.*

The patient was a female, aged 34. There was a pulsating tumour occupying the inferior third of the neck, above the right sterno-clavicular articulation. The bruit de soufflet was heard distinctly over the chest, but more remarkably on the *left* side and over the *left* clavicle. The arteries of the *right* arm beat fully and strongly, those of the left comparatively feebly. The enumeration of the general symptoms is unnecessary, as they presented no peculiarity.

The patient died in three weeks after her admission into the hospital.

*Dissection.* If the heart presented any alteration, it was smaller and firmer in structure than natural.

The ascending portion and the arch of the aorta greatly dilated; its lining membrane inflamed, of a deep red colour, and easily detached from the fibrous coat underneath. Between these structures there were numerous specks or patches of a soft and white deposit, which separated with the lining membrane on tearing it off. The fibrous coat was also evidently inflamed, and of a rose pink colour.

Where the arteria innominata is given off, but distinct and separate from it, the communication existed between the aorta and the aneurismal tumour. It was sufficiently large to admit the little finger, round, perfectly smooth, and apparently covered by a prolongation of the lining membrane. The sac seemed to have been formed by a dilatation of all the coats of the aorta at this particular spot, and to determine the question as to its being *really a true aneurism*, the structures were accurately examined. The deposit already mentioned, as lying under the lining membrane of the aorta, was also seen throughout the entire extent of the sac, indicating that the same tissues existed in both, and that the aneurism was formed of a distended portion of the aorta apparently deprived of its elasticity."

The preceding cases are interesting to those who look upon Nature with a curious eye, and, comparing symptoms with structural changes, investigate the order of cause and of effect. For our own parts, we confess that we prize such facts more highly than those laboured and ingenious speculations, which distract while they delight, and display the buoyant levity of fancy, rather than the solidity of matured understanding.

The object of Mr. Porter, in his remarks upon the cases, is, apparently, to attribute the occurrence of aneurism to loss of elasticity in the tunics of the artery, and that loss of elasticity to inflammatory action. The first of the positions will scarcely be disputed, but a glance at the proofs of the last may be permitted. Those proofs are of a double order—one depending upon symptoms—the other upon cadaveric alteration.

The proof from symptoms may be stated to be this—"the pains experienced previous to the development of those symptoms, which may be considered as more immediately occasioned by the aneurismal tumors." Did we feel inclined to trust our own observation and experience, we should say that the pain to which Mr. Porter has referred, has seldom existed prior to the commencement, or even in the early stage, of aneurism, but rather when the tumor has begun to encroach upon contiguous parts. Let us look at the phenomena of his own facts.

In the first case, the patient was attacked with pain *eighteen* days before his decease. On dissection, there were found three aneurisms, one of which had increased to such a size as to open by ulceration into the œsophagus, and to exercise a pressure on the right bronchus.

Does Mr. Porter believe that this was the product of eighteen days?

In the second case, there is no mention of the existence of the symptom.

The same remark is applicable to the third case.

In the fourth case, the narration is too ambiguous to allow any certain conclusion to be drawn. It is stated that a pulsating tumor had been noticed for a month, and that pain had been experienced from the commencement of the disease. If this and the appearance of the external tumor are intended by Mr. Porter to be considered synonymous, we think that the justice of the inference might be doubted.

From this critical review of the cases themselves, it may appear to the experienced portion of our readers, that the presence of pain is inconclusive evidence of the existence of inflammation. The second argument employed by Mr. Porter is the pathological condition of the vessels.

We may refer to numerous papers in this Journal, the object of which is to shew the difficulty involving the distinction of staining of the inner tunics of an artery from genuine arteritis. When, therefore, Mr. Porter appeals to the redness of those tunics as a proof of inflammatory action, we feel that it is almost equally impossible to agree with, or to differ from him. The prevalence of atheromatous depositions, and even ulcerations of the internal coat, may display the existence of inflammation as a consequence, but they do not satisfactorily establish its precedence as a cause.

In conclusion, we would observe that the symptoms and the nature of idiopathic arteritis are at present shrouded in obscurity. But it is not advisable to endeavour to remove it by hasty and by bold assumptions, which always confer additional perplexity on the questions they are intended to unravel and elucidate.

### II. Cases of Amputation in Spreading Traumatic Gangrene.*

We need scarcely inform the intelligent reader, that the propriety of amputating during the spreading of traumatic gangrene has excited, within these last few years, much difference of opinion among surgeons of talent and experience. Mr. Porter, actuated by a laudable desire to assist the profession in arriving at some rational and definite conclusions, has related in our excellent contemporary, some cases that occurred in the hospital to which he is attached.

The practice in question has been adopted in the Meath Hospital since the year 1818, and the facts will serve to shew, that the operation is not so fatal as has been usually imagined, and that, where it has failed, this has not been the effect of the invasion of the stump by mortification. Without any farther preliminary observation, we shall pass to the details of the cases before us.

Case 1.—*Mortification of the Leg, from Rupture of the Posterior Tibial Artery—Operation unsuccessful.*

"March, 1820.—A man applied among the externs at the Meath Hospital, complaining of deep and intense pain in the right leg, occasioned by a severe blow inflicted on the back of it. On examination, there was no appear-

---

* Ibid.

ance of injury, no swelling, no discoloration, nor even tenderness on pressure. He scarcely exhibited the smallest lameness, and his case attracted very little attention.

Displeased at this neglect, he applied at other hospitals, at some of which he was regarded as an hypochondriac, and at none was the case deemed to be of importance.

About six weeks after his first application at the Meath, he felt something give way within the leg, which immediately began to well. In the course of a few hours it had attained a great size, was pale, shining, and tense, yet retained the impression of the finger on pressure. His pulse very small and quick, tongue brown, he was thirsty, and had vomited several times, his face was pallid, and he had a wild, yet dejected expression of countenance. He was admitted into the hospital under the care of the late Mr. Thomas Roney.

On the following day matters had assumed a very different appearance; the leg was livid, cold, and insensible; the skin had burst, and a large coagulum of dark blood protruded; the lower half of the thigh was mottled, of a dark amber or brown colour, and emphysema had extended nearly to the groin. He acquiesced at once in the proposal of having the limb removed, which was performed by Mr. Roney, but the result was not fortunate. The irritative fever still continued; the stomach rejected every thing both of food and medicine, and he sunk and died about 36 hours afterwards."

The stump exhibited no appearance of gangrene.

On examining the limb the posterior tibial artery was found ruptured, and a large quantity of blood lay under the deep fascia. In this a ragged sloughy aperture was seen, and the limb was in all directions extensively injected with blood.

As Mr. Porter justly observes, it seemed as if the artery had been injured by the original blow, and had bled under the fascia, which subsequently gave way and burst, when mortification rapidly ensued.

In the second case the mortification succeeded to compound fracture of the leg. It appeared on the third day, and displayed the phenomenon of a line of blue vesication round the edges of the wound. On the eleventh day this was thrown off as a slough, and the broken bone was exposed. On the thirteenth day another line of vesication appeared. In the night the bad symptoms of vomiting, restlessness, and incoherence supervened. By the morning the mortification had extended for upwards of a hand's breadth, and the lower third of the thigh was discoloured. The general symptoms were those of prostration.

Amputation was now performed. He fell into a state of low muttering delirium, and died in twenty-eight hours.

The stump displayed no peculiarity of appearance.

CASE 3. *Fungous Tumor of the Periosteum—Gangrene—Amputation—Recovery.*

A labourer, æt. 42, admitted May 29th, 1833, under the care of Mr. M. Collis.

The middle of the leg was occupied by a tumor, apparently deep-seated, and from this a fungus, the size of a walnut, protruded through an incision which had been made. The tumor had existed for eight months;—the incision had been practised two months prior to admission.

On the fifth day after his reception, the fungus had increased to an enormous size, and looked black and sloughy. The discharge was offensive and profuse. The limb as high as the knee was mottled and œdematous. On this day, amputation was performed.

No attempt at primary union was observed, and for three weeks the man was in a critical condition. He required support by wine, and opium, and bark. In July an abscess was opened on the outside of the thigh, and this continued discharging for a month. The stump then slowly healed, but the patient was unable to be dismissed from the hospital till September.

CASE 4. *Compound Fracture—Gangrene—Amputation—Rapid cure.*

"June 5, 1833. John Reilly, æt. 30, a servant, received a compound fracture of the leg by a fall from a horse; the bone protruded considerably, and there was smart hæmorrhage. The fracture was reduced on the spot, the limb bandaged, and he was conveyed to hospital, a distance of ten miles. Notwithstanding the utmost care and the most judicious treatment, gangrene took place on the 13th, the leg was enormously swollen, very tense, of a dark purple colour, and mottled almost to the groin. The foot was swollen, œdematous, and cold; the wound in a state of slough. The operation of amputation was performed by Mr. Roney very high up above the knee, but so rapidly had the disease spread, that the incision was made through cellular tissue, loaded with a reddish serum. It is unnecessary to detail the daily reports of this case, which went on more prosperously than happens in the majority of similar cases. An abscess formed on the right side of the stump on the fifth day after the operation. There never was any appearance of union by the first intention, but the stump granulated well, and healed kindly from the bottom. When discharged from the hospital, on the 12th of August, (within two months after the limb had been removed,) the stump was completely healed, and the bone admirably well covered."

Case 5. *Simple Fracture of the Leg —Gangrene—Amputation—Death from Phlebitis of the Stump.*

The patient, a powerful man, received a severe simple fracture of the leg, in a state of intoxication. Two days afterwards, the limb was enormously swollen, tense, and elastic—its colour, as far as the knee, of a deep brown, and the foot quite cold. Mr. Porter performed amputation. The following are the notes of the examination of the limb.

"There was an oblique fracture of the tibia fully six inches in length, terminating in a very sharp point, about four inches above the ankle joint, and comminuted fracture of the upper end of the fibula, where it was articulated to the tibia. The limb generally was gorged with blood, the fibres of the gastrocnemius and soleus muscles being separated into small bundles by the infiltration. A vast quantity of blood also lay between the soleus and the fascia that lies behind the posterior tibial artery, the source of which could not be discovered by injecting water into the vessels. The anterior tibial nerve, where it passes round the head of the fibula, was completely torn through. The cellular tissue of the limb was loaded with a semigelatinous serum of a red colour. The* knee joint was completely filled with a fluid blood, although there was no lesion of the synovial membrane."

Symptoms of low fever, and of great irritability, set in on the fourth day. On the 8th of February the patient died.

*Dissection.* Inflammation, with the presence of lymph and of pus in the femoral vein. A collection of fetid purulent matter on each side of the bone. No attempt at granulation.

No mention made of the condition of the viscera.

Case 6. *Compound Fracture of the Leg — Gangrene — Amputation — Recovery.*

Anne Reynolds, æt. 27, admitted Feb. 27th, 1832, under the care of Mr. M. Collis.

There was a compound fracture of the right leg, with protrusion of the bone about a hand's breadth above the ankle. On the fourth day the foot and the leg below the wound were insensible and cold—the edges of the wound were of a dark and livid tint—the discharge was profuse and abominably fetid. The leg to the knee was mottled, of a dark brown colour, œdematous, and boggy.

* "I have before me the notes of a case of compound fracture, in which a similar effusion appeared in the knee joint. Amputation was performed after gangrene took place, and the patient recovered. I do not, however, think myself at liberty to notice the case further, as it occurred in another hospital."

Amputation was performed by Mr. Collis. The stump was very dry, scarcely a drop of blood even oozing from its surface.

Omitting the precise characteristics of the fracture, we may state that it extended into the ankle-joint. Very fetid pus surrounded the broken extremities of the bones. The subcutaneous cellular tissue was infiltrated with air and reddish-coloured serum, as high as the tibial tubercle.

The stump for some days appeared to do well. On the seventh it opened in its entire extent, discharged a quantity of very fetid pus, and it became evident that abscesses were formed. This discharge continued some weeks, and granulation proceeded but slowly. She was supported by broths and a liberal use of wine, and took quinine and opium. The wound was skinned over on the 28th April, and she left the hospital well on the 6th May.

Case 7.—*Popliteal Aneurism—Ligature—Secondary Hæmorrhage—Gangrene—Amputation—Recovery.*

A man, æt. 37, was admitted on the 7th of August, 1831, with popliteal aneurism in the left ham. On the 13th, the artery was secured in the upper third of the thigh. On the 22d, blood issued from the wound, and five or six ounces were lost, before cold applications succeeded in arresting the hemorrhage. The bleeding recurred two or three times, when the wound was opened to the bottom, and a graduated compress introduced, and secured by an instrument of Mr. Crampton's, which pressed upon the compress without interfering with the venous circulation of the limb. The hæmorrhage was stopped, and the ligature came away on the 1st of September. The patient left the hospital with the wound quite healed, but complained of pain in the sole of the foot, the instep, and the heel, which was only relieved by gentle friction.

On Oct. 8th, he was re-admitted for swelling and stiffness of the knee, attended with intense pain, which were removed by the use of calomel and opium. Two abscesses successively formed in the leg, and on Nov. 9th, a dark-coloured spot appeared on the dorsum of the foot. On the 15th, the greater part of the foot, the great toe, the second, and a portion of the third, were affected with mortification. The remaining toes were of a dull purple colour at their extremities, and he complained of intense pain. From the roots of the toes to the instep there was a patch of a light brown colour, dry, and apparently below the level of the surrounding parts, which were œdematous, swollen, and deprived of their cuticle. The œdema reached to the knee. Amputation was decided on, and performed in the usual manner high up in the thigh.

The stump granulated kindly, and the patient left the hospital, cured, in six weeks.

Observations would rather tend to diminish than enhance the value and the force of the preceding facts. If a surgeon exists, whose prudence or whose fears have induced him to refuse to a patient affected with traumatic gangrene, the chance that an operation could present, we earnestly entreat him to read, to reflect, to be convinced, and to discard his fatal hesitation.

An exception may be taken to the term traumatic gangrene, or spreading mortification. A cursory glance at even the few cases adduced by Mr. Porter, will readily demonstrate that all are not of a similar description. In the last of those cases, a *black* spot appeared upon the dorsum of the foot, and six days elapsed before the instep and the toes were sphacelated, whilst œdema extended no higher than the knee.

How different are these characters from those of the fifth case. In that, a great amount of mischief had arisen in the space of two days from the reception of the injury. The limb was then found to be enormously swollen, elastic, tense, and of deep brown colour as high as the knee-joint. On dissection, the cellular tissue was loaded with a red semi-gelatinous serum. In the sixth case, the "gangrene" was fully established on the fourth day after the accident—the subcutaneous cellular tissue was infiltrated with a reddish-coloured serum and with air—the bones were bathed in a fetid pus.

A little consideration will point out, that the majority of these cases were instances of diffuse *inflammation* of the cellular tissue, terminating with rapidity in its destruction, and in that of the integuments. The last case on the list of the candid and intelligent author of the paper is really one of sphacelus, probably dependent on deficient circulation. We differ from Mr. Porter in deeming this more formidable and more unfavourable than the former.

The length to which this notice has extended, is a proof of the importance we attach to the subject and the cases. We again recommend the latter to the serious attention of practical surgeons.

---

### VIII. Lock Hospital.

### Some further Remarks on the best Mode of treating Gonorrhœa.

[By H. J. Johnson, House-Surgeon.]

In the last Number of this Review, I took the liberty of reporting some cases of gonorrhœa, and of offering a few remarks on that affection and its consequences. I suggested that the treatment commonly employed might be found, on examination, too stimulating in its character and too unfortunate in its results; and I proposed a plan which appeared more consistent with our ordinary principles of therapeutics, and seemed more successful when submitted to the test of practice. Since those observations were written, an additional, indeed an enlarged, experience has tended to convince me of the correctness of my opinions, and enabled me to add to the force, as well as to the number, of my statements.

I am satisfied, in my own mind, that the ordinary method of treating gonorrhœa is injurious and unsafe. When we look at the practice recommended and adopted, we may readily discern two opposite extremes, which seem to be surrounded with danger or with inefficiency. On the one hand, we perceive the almost indiscriminate exhibition of stimulants—cubebs, copaiba, turpentine, injections—on the other, the use of salines and antiphlogistic medicines. The former are constantly productive of immediate inflammatory complications—of chronic inflammation of the corpus spongiosum or the lacunæ—or of that intractable affection, long-continued pain in micturition, with little or with no discharge. The latter class of remedies are insufferably slow in removing the acute stage of gonorrhœa, and appear to favour, in a material degree, the subsequent occurrence of gleet.

Perhaps I may be thought censorious in my condemnation of these contrary methods of treatment. I would not willingly incur, or at least deserve, the imputation, and, therefore subjoin the particulars of some cases calculated to display their injurious effects. My limited space will prevent me from detailing more than two or three.

Case 1.—*Acute Gonorrhœa and Inflammation of the Corpus Spongiosum, treated by Turpentine.*

Thos. Harrison, æt. 42, a harness-maker, was admitted an out-patient Dec. 13th, 1833.

He had yellow, profuse, and thick discharge—pain in micturition and on pressure, experienced along the urethra as far back as the anus—slight chordee—some induration, and tenderness on pressure of the corpus spongiosum. The bowels were freely open.

The complaint had existed for six weeks, and was worse rather than better. He had been treated by pills, containing turpentine, which, he said, "had very nigh killed him." So wroth was he against the person who prescribed the pernicious medicine, that he regretted he was not sufficiently rich to enjoy the luxury of prosecution.

I cupped this patient on the perinæum—and prescribed pills of calomel, with opium and emetic tartar at night, a draught of senna and of colchicum each morning, and diluents, with tragacanth, nitre, carbonate of soda, and Dover's powder during the day. He returned on the 17th. The pain in micturition and on pressure had almost vanished—there was no chordee—and the discharge was thinner, and diminished in quantity. This rapid and marked amendment leaves no doubt on the mind of the reflecting surgeon, of the propriety of the practice under which it was observed.*

CASE 2. *Acute Gonorrhœa—Inflammation of Corpus Spongiosum—Bloody Urine—Pyrexia—treated by Turpentime.*

Charles Haisman, æt. 36, a smith, admitted an out patient, Dec. 10, 1833.

Yellowish discharge—pain in micturition, and after it, felt along the inferior surface of the urethra—tenderness in the same situation on pressure—induration of the corpus spongiosum—micturition itself frequent — bright blood occasionally mingled with the urine—pains in the loins. Tongue white and loaded — bowels confined — some pyrexia.

Complaint has existed for three months. The medical gentleman under whose care he has been, and whose last prescription he has brought with him, has ordered, and the patient has been taking, for the symptoms above mentioned, pills of sulphate of zinc and of turpentine.

I prescribed the same remedies for this as for the other patient.

On the 14th the urine was free from blood, but pain was still experienced in the loins and the urethra.

*C. c. lumbis ad* ℥viij. *Hirud.* x. *peni. P. c̄. omnibus.*

17th. Only very slight pain in micturition—discharge whitish, and diminished in its quantity. Other unpleasant symptoms gone.

*Rep. Hirud.* viij. *peni.*

The same observation seems applicable to this as to the former case.

CASE 3. *Acute Gonorrhœa with Inflammation of the Glans—treated by Turpentine.*

George Bowen, æt. 35, admitted an out patient, Dec. 17, 1833.

Profuse thin yellow discharge—pain in micturition experienced at the orifice—vascularity and inflammation of the surface of the glans. Tongue white and loaded.

Complaint has existed for three weeks and a half. For the first eight days he had no pain. He has been under the care of an eminent surgeon in Westminster, who has treated him by pills of turpentine. Is getting worse.

I ordered the patient calomel at night —senna with colchicum in the morning—and diluents with the combination of gum and of nitre, to which I have sufficiently alluded.

These cases have been mentioned because they have occurred within the last few days, and are not inappropriate illustrations of the extraordinary notions that would seem to be entertained of the nature of gonorrhœa and the principles of treatment. It may be urged that they are picked and unfrequent examples of the failure of stimulating remedies. I might deluge these pages with facts in refutation of such an objection. I cannot resist the advantageous opportunity of noticing a few, and for a double purpose:—to display the ill effects of the methods condemned, and the benefits derived from that recommended. The cases which have just been related are not sufficiently advanced to confirm the success of the latter.

CASE 4. *Long and ineffectual Use of Cobaiba, &c.*

A baker, aged 22, applied to me three days ago with yellow and copious discharge—pain in the part of the urethra contained within the glans—and painful nocturnal erections. The tongue was loaded and the bowels were confined.

These symptoms had existed, better or worse, for the period of six months. The treatment had consisted of the exhibition of copaiba, injections, and cubebs, during the existence of pain and inflammation.

I ordered calomel, senna, and diluents, and applied a blister to the penis. The patient returned to shew himself to-day. The pain is nearly gone.

* These observations being written on this day (the 17th), it is of course impossible to give the sequel of the case.

Case 5. *Acute Gonorrhœa—Inflammation of Corpus Spongiosum, &c. treated by Cubebs.*

A patient applied to me on the 7th of this month with symptoms of acute gonorrhœa—inflammation and induration of the corpus spongiosum—painful erections and chordee—paraphymosis, with thickening and transverse ulceration of the prepuce—and some pyrexia.

The complaint had existed for two months—the paraphymosis had originated in manual retraction of phymosis. The patient had been treated throughout by cubebs.

I have ordered calomel, senna, repeated blisters, and diluents. The patient is materially relieved.

Case 6.—*Discharge with pain for seven months—cured in one month.*

Geo. Ash, æt. 23, a tailor, admitted an out-patient, October 12th, 1833.

Thin yellow discharge—some pain an inch and a half behind glans—tenderness in that situation on pressure.

These symptoms have existed since March last, a period of seven months. Had great pain and chordee at first. Has been treated by cubebs and copaiba.

*Hyd. sub.* gr. v., *Opii,* gr. ½, *o. n.*

*Hs. sennæ,* c̄ *Vin. colchici,* ʒj. *o. m.*

*Hirud.* viij. *peni.*

19th. No pain whatever for the last three days. Observed only "the slightest discharge" yesterday. Gums tumid.

*Bals. copaib., Tinct. catechu,* āā ʒss. *bis die. Omit. pilulæ.*

22d. Scarcely any discharge.

*P.* c̄ *Bals. ter die. et haust. sennæ.*

*Inject. Plumb. acet.* gr. ij. *ad* ℥j. *bis die.*

29th. No discharge for two days.

*Aug. Inj. ad* gr. iv. *ad* ℥j.

*Omit. haust. sennæ.*

Nov. 2d. *Aug. Inj. ad* gr. vj. *ad* ℥j.

Nov. 12th. Has had no return of discharge since the last report. To have injection sufficient for a month. Dismissed cured.

Case 7. *Acute Gonorrhœa, with Phymosis—cured in less than a month.*

Richard Draper, æt. 17, a baker, admitted an out-patient, Oct. 8th, 1833.

Phymosis—profuse yellow discharge—much pain in micturition. Pulse frequent—bowels confined.

The gonorrhœa has existed for seventeen days—the phymosis for two days. Seems to have taken copaiba. It made him much worse.

*Hyd. sub.* c̄ *Opio et Ant. tart. o. n. in* iv. *vices.*

*H. salin.* c̄ *Vin. ant. tart., Mag. sulph. et Vin. colch.* 4*ter die.*

*Dec. hord.* c̄ *Tragac. pro potu.*

12th. No pain for two last days—phymosis less—discharge copious.

*Omit. pil. et haust.*

*Haust. sennæ* c̄ *Vin. colch. o. m.*

*Pulv. trag. c.* c̄ *Pot. nit. &c. ter die.*

*Bals. copaib.* c̄ *Tr. catechu, o. n.*

19th. Swelling gone—discharge very greatly diminished—prepuce still tight, and evincing a disposition to superficial excoriation.

*P.* c̄ *Bals. bis die.*

*Inject. Plumb. acet.* gr. ij. *ad* ℥j.

29th. No discharge for last six days. Phymosis gradually reducing—some thickening of prepuce left.

Nov. 6th. No return of discharge or pain. Dismissed cured.

Case 8. *Discharge for eight months—cured in less than one month.*

Thos. Everard, æt. 38, a wire-drawer, admitted an out-patient, Aug. 26th, 1833.

Yellow thickish discharge—no pain.

This has existed for eight months. It began with acute gonorrhœa. He has been treated by cubebs, copaiba, &c. His wife has been infected by him.

*Hyd. sub.* gr. iij. *o. n.*

*Magnes. sulph.* ℥ss. *o. a. m.*

30th. Discharge increased with scalding.

Sept. 3d. Very little scalding now—painful erections at night, the pain being experienced at the orifice of the urethra. Gums slightly affected.

*Ext. conii,* gr. x. *o. n.*

*P.* c̄ *Mag. sulph.*

*Omit. pil.*

9th. Scalding and painful erections gone—discharge much less; yellowish and thin.

*Inj. Dec. papav.* c̄ *Plumb. ac.* gr. j. *ad* ℥j. *o. n.*

*Conf. sennæ* c̄ *sulph. o. m.*

14th. Discharge almost gone.

21st. Merely a very slight discharge in the morning; it is yellowish. A little pain when the penis is erect.

*P.* c̄ *inject. bis die.*

Oct. 5th. Discharge has not appeared for a fortnight.

Nov. 5th. Has remained cured.

Case 9. *Discharge for three months—cured in a few days.*

Geo. May, æt. 19, admitted an out-patient, Dec. 6th, 1833.

This patient had vascular warts on the glans and on the prepuce. These had existed for five weeks. He had also urethral discharge. This had continued for three months, and had been attended in the first instance with pain. He had several times had gonorrhœa previously. He had only taken some salts for this.

I ordered calomel at night and senna in the morning, with an injection of the acetate of lead thrice daily. The discharge disappeared on the next or the following day, and it has not subsequently returned.

Case 10. *Discharge for ten months—cured in a week.*

Robert Skelton, a tailor, æt. 26, admitted an out-patient, Oct. 11th, 1833.

Discharge yellow and not profuse—no pain. Has had the discharge for ten months.

*Pulv. rhei* c̄ *Magnes. o. n.*

*Haust. sennæ* c̄ *Vin. colch. o. m.*

*Inj. aluminis,* gr. ij. *ad* ℥j. *bis die.*

14th. No discharge observed to-day.

21st. No return of discharge.

Ordered enough medicine for a fortnight. To return, if the complaint should re-appear. Cured.

Case 11. *Discharge with pain for two years—cured in less than two months.*

Thomas Delaney, æt. 25, a shoemaker, admitted an out-patient, Oct. 11th, 1833.

Whitish discharge—pain felt near the site of the frænum.

Complaint has existed for two years. Has taken copaiba, and used a great variety of treatment. Five years ago he had his first gonorrhœa. This was treated in the acute stage by copaiba and the spiritus ætheris nitrici. Inflammation of the testicle was the consequence,

*Hyd. sub.* gr. iij. *o. n.*

*Haust. sennæ, o. m.*

*Hirud.* xii. *peni.*

21st. Pain nearly gone.

*Emp. canth. penis infer. parti.*

28th. Pain gone.

*Rep. pil. et Haust. et Emp. Canth.*

*Pulv. Tragac. &c. bis die.*

Nov. 4th. Some tenderness in the testis formerly affected—some hardness of the epididymis.

*Hirud.* x. *testi. P. suspensorium.*

20th. Affection of testis passed away in a few days—no pain in micturition. Discharge whitish, thin.

*Inj. Pl. acet.* gr. iij. *ad* ℥j. *ter. die.*

*Bals. copaib.* c̄. *Tr. catechu, o. m.*

*Pulv. Rhei,* c̄ *Magnes. o. m.*

*Omr. alia.*

Dec. 3d. This patient has for some time been free from any discharge.

Case 12. *Acute Gonorrhœa for two months—cured in one month.*

Charles Daw, æt. 23, a butcher, admitted an out patient, Sept. 27, 1833.

Much thin and yellow discharge—a great deal of pain along the course of the urethra.

Complaint has existed for 2 months. Has taken copaiba.

*Hyd. sub.* c̄ *Op. et Ant. t. o. n. in vices tres.*

*H. sen.* c̄ *Colch. o. m.*

*Pulv. trag. &c. 4tis horis* c̄ *Dec. hord.*

Oct. 4th. Pain much diminished—very little discharge.

*Emp. Canthar. peni.*

11th. Pain less—felt only at the orifice—latter vascular.

18th. Discharge gone for a week—still slight pain in micturition.

26th. Discharge returned this morning—it is yellowish—very trifling pain in the site of the frænum.

*Hirud.* vi. *peni.*

*P.* c̄ *Pulv.*

*Bals. copaib.* c̄ *Tr. catechu, o. n.*

28th. No pain.

*P.* c̄ *Bals. bis die.*

*P.* c̄ *H. sennæ, o. a. m.*

*Omr. alia.*

Nov. 2d. No discharge for a fortnight. This patient subsequently reapplied with catarrhal affection. He had had no return of the discharge.

Case 13. *Discharge with Itching of the Thighs for four months — cured in four or five weeks.*

A gentleman was recommended to my care by a friend, and first consulted me five or six weeks ago.

The prepuce was swollen, œdematous, and slightly inflamed—there was yellow urethral discharge—there was no pain in micturition or after it. There was a peculiar itching on the inside of each of thigh. The tongue was loaded —the bowels irregular. This gentleman had lived freely, and drank much port-wine.

The discharge had existed for four months, and the itching had been the first and the constant symptom. There had been some pain in the commencement, but it had never been severe. The patient had been treated by copaiba, with which, and with its ill success he was utterly disgusted.

I prescribed calomel at night, and a mixture containing the carbonate and sulphate of magnesia in the morning. I directed the application of a lotion of the acetate of lead to the prepuce, and its subsequent use as an injection.

The swelling of the prepuce rapidly subsided, and the urethral discharge was speedily arrested. After a week's absence it returned, as the consequence of a port-wine debauch. A little more active purgation, the cessation of the employment of the injection for a day, and its repetition, with more attention to regimen, were again effectual in checking the discharge in the course of a very few days. When I saw the patient last, the discharge had not been observed for ten days, the sensation of itching of the thighs was nearly gone, and the general health was greatly improved.

Case 14. *Discharge with slight Scalding for five months—cured in a fortnight.*

W. B. æt. 22, a groom, admitted an O. P. Sept. 27, 1833.

Yellow discharge thin—a little scalding.

Complaint has existed for five months —has taken much copaiba, &c.

*Hyd. sub.* gr. iij. *o. n. in noctes tres.*

*H. Sennæ, o. m.*

*Bals. copaib.* c̄ *Catechu, bis die.*

Oct. 11th. Discharge much less—very slight scalding still in glans.

*Hirud.* viij. *peni.*

21st. No pain since the application of the leeches — no discharge for ten days.

28th. No return of discharge. Cured.

I might mention other cases of this description, but I am impelled by two contrary motives to stop and to proceed. If only a few instances of success were related, the reader might suppose that I put my best foot foremost;—if a numerous array were advanced, he would feel disgusted with their tiresome monotony. Perhaps the striking nature of those which have been stated may render an addition to their number unnecessary. When a certain plan of treatment has long been pursued without effect, or without success, and another method, of a contrary description, has been speedily attended with the happiest results, I conceive that his mind must be formed of uncertain materials indeed, who can pause in determining which to prefer. It must not be forgotten that during the experiment the external circumstances remained the same. The patients were *not* received into the house, supplied with wholesome and with proper food, debarred from indulgence in excess, and preserved from the necessity of toil, but they still pursued their usual occupations, and were still exposed to all the influences that might aggravate or perpetuate their symptoms. To say more would be superfluous, to say less would be unfair. The comparison and the conclusion must be left to the candid and considerate reader.

The cases brought forward have been chiefly those in which the disease had existed for some time, and in which other treatment had proved ineffectual. It may not be improper to relate two or three in which the plan it is the object of this and of a former paper to display, was adopted in an earlier stage. The authentic particulars of special facts are more fatiguing to the mind than general inductions, but the strict inquirer of the truth of what he reads, remains unsatisfied and unconvinced without them.

CASE 15. *Discharge without pain speedily cured.*

Michael Connor, æt. 36, a servant, had connexion a day or two before the 29th of October last. On that day he applied to me. There was yellow urethral discharge, *without* pain. He said that copaiba always cured him.

He was ordered copaiba and catechu twice daily, and the discharge was arrested on the following day. He was perfectly cured.

CASE 16. *Gonorrhœa treated on the first day—cured in four or five.*

A young gentleman engaged in the study of the law, applied to me on the 29th of October last, in a state of considerable alarm.

He had had connexion five days previously, and on the morning of his application he had noticed some discharge accompanied with tingling, scarcely amounting to pain, in the act of micturition. He was florid and robust—accustomed to smoke much and to drink a *little*.

I ordered the pill of calomel, opium, and tartar-emetic at night for three nights—the draught of senna and colchicum each morning—the powder of tragacanth, &c. four times daily, with abundance of barley and toast-water—repose—and low diet.

Nov. 5th. Discharge ceased in a day or two. Has still some tingling, and even pain, at the orifice in the act of micturition.—*P.* c̄ *Haust. et Pulv.*

10th. Has had no symptom since the 6th.

13th. Remains well.

I have frequently seen this gentleman since. He continues free from complaint.

CASE 17.—*First Acute Gonorrhœa—cured in three Weeks.*

A gentleman, æt. 21, applied to me on Oct. 10th, 1833, with thick copious discharge—much scalding in the act of micturition—painful nocturnal erections, the pain being felt at the orifice, and so severely, that he was generally obliged to leave his bed. He was healthy, but of rather a strumous constitution.

This was his first gonorrhœa. He had noticed the discharge two days. He had had connexion one week before he observed the discharge.

*Calomel, &c. every night for four nights.*

*Tragacanth, &c. four times daily in barley-water.*

*Senna, &c. every morning.*

*Ten leeches to the penis.*

16th. Pain and painful erections gone. Discharge thinner—free.

*Pulv. Rhei,* c̄ *Magnes. o. mane.*

*Bals. copaib.* c̄ *Catechu, o. n.*

*P.* c̄ *Pulv.*

*Omr. Pil. et Haust.*

18th. *P.* c̄ *Bals. bis die. P.*

23d. Discharge very much diminished. Balsam makes him very sick.

*Interm. Bals.*

*Inject. Plumb. acet.* c̄ *Dec. papav.* gr. ½ *ad* ℥j. *ter. die.*

*Pulv. rhei.* c̄ *Magnes. p. r. n.*

27th. *Utatur Inject. 6ties indies* (gr. ij. *ad* ℥j.)

31st. No discharge since yesterday morning.

Nov. 13th. No return of discharge. Has continued to use the injection and to abstain from wine and from malt liquor.

The diet of this gentleman was not strictly antiphlogistic, in order to avoid the risk of suspicion. He took less meat than usual, and chose fish when the opportunity of choice was presented. He drank no wine nor beer, and took as little exercise as possible.

CASE 18. *First Acute Gonorrhœa—arrested in a month—slight subsequent Relapse—cure.*

A gentleman, æt. 24, applied to me on the 11th of November last.

Thin, yellow, and profuse discharge—pain and scalding at the orifice in micturition. No pyrexia. Of a strong constitution, and not very intemperate, though an Oxford man. Has been for many years exposed to infection in Oxford, in London, and in Paris. Never contracted the complaint before.

Had connexion with a stranger, his usual acquaintance being out of town, about ten days ago. Two or three days ago he thought he observed for the first time discharge and scalding. Has taken some salts.

*Cal.* c̄ *Ant. tart. et Op. o. n. in noct.* iij.

*H. Senn.* c̄ *Colch. o. m.*

*Tragac. &c. in dec. Hord. 4ter die.*

*Low diet and diluents.*

Nov 15th. Discharge abundant and thinner—very little pain at the orifice—mouth apparently a little affected—bowels freely opened.

*Omr. Pil. et Haust. sennæ.*

*P.* c̄ *Pulv.*

*Pulv. rhei,* c̄ *Magnes. o. m.*

*To eat some fish.*

19th. Less pain at orifice—otherwise much the same—pale œdema of the prepuce.

*Resum. H. senn. vice Pulv. rhei.*

*Lavatio frigida.*

26th. Still very slight pain at the orifice, and indeed along the urethra—discharge less—œdema diminished.

*P. Hirud.* x. *peni ac perinæo.*

29th. Very slight pain at orifice, amounting to little more than tingling. Was relieved by the leeches.

Dec. 1st. Pain gone—some itching in the urethra—discharge thin, yellowish—œdema gone.

*Bals. copaib.* c̄ *Catechu,* ʒj. *o. n.*

*Rep. H. senn. o. a. m.*

*P.* c̄ *pulv.*

3d. Discharge almost gone. Itching less.

*Rep. Bals. bis die.*

5th. Discharge gone. Itching has ceased.

*Inject. Plumb. ac.* gr. j. *ad* ℥j. *bis die.*

*Rep. H. senn. o. 3tio mane.*

12th. Has rather indulged in his eating lately, although he has avoided stimulating fluids. Has had for the last two or three days and nights almost continued erections with emissions. Has become very fat.

Last night he observed slight discharge, which this morning is yellow and distinct. Has omitted the copaiba for the last few days, and only employed the injection.

Having remarked, on more than one occasion, that the discontinuance of purgatives, or, what may in some measure be deemed equivalent, the resumption of more nutritious or more stimulating diet has been succeeded by a marked disposition to erections and emissions, and that these in their turn had appeared to occasion a return of the discharge, I determined again to deplete this gentleman. His increased obesity and remarkable salacity made me think that the discharge depended on augmented fulness of the vessels.

I ordered him to live on fish—to repeat a strong purgative draught every morning—and to intermit the copaiba and injection for a day.

On the following morning the discharge was diminished, and the active purgation of only one day had impaired the disposition to emissions.

He was ordered to return to the injection and copaiba, the purgative draught and fish diet being persisted in.

In three or four days, the discharge had completely disappeared, and it has not again returned. The patient was directed to continue his precautions with respect to diet, and pursue the same plan, with reference to medicine, for the period of three weeks or a month from the cessation of the symptom.

I might mention other cases of this description; but I fear I should fatigue the most patient reader. It is sufficient to observe that they illustrate the same facts and display the same characters. In none was copaiba given, nor were injections used, till pain in micturition or in erections had vanished, and in all a moderate purgation was employed. The patients were cured in from three to five weeks; and, as I am now speaking of inflammatory gonorrhœa, I conceive that this success is of no despicable nature.

It was stated, in a preceding portion of this paper, that saline medicines appeared to favour the subsequent occurrence of gleet. Probably a general expression of experience would be preferable, in this instance, to particular details. I have merely to observe that, in the only *two* cases of this troublesome symptom that have occurred under my management of gonorrhœa, the treatment in the commencement mainly consisted of salines. Whether this post hoc argument is conclusive, may safely be left to the determination of others; but the impression on my mind against these medicines is so strong, that I never venture to prescribe them.

A rapid review of the principles of treatment laid down, and the items pursued and recommended, may not be totally irrelevant.

The object of the author of this paper is to shew that gonorrhœa, though, perhaps a specific complaint, is commonly attended with the symptoms, and frequently complicated with the ordinary consequences, of inflammatory action. This inflammation may be limited to one tissue, or extend to several. In the commencement, it is seated in the mucous membrane; if aggravated, it invades the corpus spongiosum, the lacunæ, the cellular membrane of the penis or the perinæum, or it spreads by continuity of texture to the prostate, the bladder, or the testicle.

The symptoms characteristic of inflammatory action are briefly and obviously these:—pain in the act of micturition or erection, experienced in both at the orifice alone, in that part of the urethra contained within the glans, or, more rarely, along a greater portion of the canal. The enlargement and tenderness of the lacunæ—the thickening and pain on pressure of the corpus spongiosum, with its usual symptom, chordee—the œdema or the fluctuating swelling of the penis—the deep-seated pain, the swelling, and the obscure fluctuation of the perinæum—and the special symptoms which attend on inflammation of the bladder or the testis, are sufficient and decisive evidence of the inflammatory complications enumerated above.

So long as there is *pain*, I conceive that there is sufficient inflammation to prohibit the internal exhibition of stimulants, or the administration of injections. If pain is removed, and lacunar enlargements or induration of the corpus spongiosum remains, I still disapprove of the use of cubebs, copaiba, injections, or other remedies of that description. Some gentlemen may entertain a contrary opinion, and triumphantly refer to their success. Without impugning their judgment or their observation, I must still presume to confide in my own. I have used the best means I possess of arriving at the truth. My field of observation has been ample, I have endeavoured to divest myself of theory and prejudice, I have carefully observed the facts which came before me, and watched the effects of methods and of medicines. If others are dissatisfied and sceptical, I can only reply, in the words of Mr. Boswell, "what won't fill a quart-pot will fill a pint," and confess myself the pint-pot in the present instance.

Gonorrhœa, then, should be treated, whilst pain remains, as another inflammatory complaint—by calomel at night—by active purgatives of senna and of colchicum—by diluting and mucilaginous drinks, combined with soda and with nitre, in order to diminish the stimulating nature of the urine*—and, if the symptoms are obstinate or severe, by leeches to the penis, or by cupping on the perinæum, succeeded, if necessary, by the application of blisters.† These means, with slight and with obvious modifications, would be such as the judicious physician would employ in the

---

* The formula into which I have *settled* is this.

*Pulv. trag. c.* ℨij. *Pot. nit.* gr. vj. *Sod. carb.* gr. x. *Pulv. ip. c.* gr. iij. *M.* 4*tis horis*, c̄ *Dec. hordei*, Oss.

Barley-water or linseed tea, or some other form of diluent, should be liberally drunk in addition to this.

† I find such benefit from leeches and from cupping, that, if the case is at all acute, I order them immediately, and repeat them frequently. The pain and scalding are speedily relieved, the period for copaiba materially accelerated, and the cure effected with more dispatch and with more satisfaction.

inflammation of any organ. Erroneous notions of the venereal disease, and the prevalent disposition of mankind to believe in a specific, would seem to have led to a general disregard of natural precaution and established principles in the management of gonorrhœa.

When pain has ceased, or when the other symptoms of inflammatory complications have subsided, then, and not till then, would I recommend the use of cubebs or copaiba. The more I see the more I am convinced that the latter is the better of the two. If the surgeon and the patient restrain their ardour, and defer its employment till the period I have specified, it is astonishing how few doses, and in what small quantity, are sufficient to diminish or to check the discharge. I am also convinced that injections of the acetate of lead or alum, but especially of lead, are infinitely preferable to others of a stimulating character. The strength of the injection of alum may be raised to the point of saturation, and that of the solution of lead to the amount of twelve or of more grains to the ounce. Copaiba need never be given in large doses—injections of lead are generally sufficient—but perhaps more caution and more discrimination are required in *timing* than in *measuring* these remedies.

There are some incidental points on which I may make a few practical remarks, or rather, on which a hint may be expended.

The florid and robust would appear to be cured with more facility than those of a pale complexion, and a nervous temperament. The symptoms of the former are often more acute but generally more amenable to measures of depletion. The latter frequently require some preparation of opium, and the best has appeared to be Dover's powder. A full dose at night, succeeded by the ordinary purgative in the morning, has relieved many urgent and anomalous symptoms. Let not the surgeon be betrayed into tonics in the treatment of these individuals. Sedatives often suit, stimulants or tonics scarcely ever.

Gonorrhœa is frequent amongst the debauched, whose constitutional powers are destroyed by intemperance and by excess. Such patients are known not to bear much depletion. But they *do* bear purging, and they bear it well. Their inflammatory symptoms are usually speedily relieved by purgation, diluents, and temperance, and their treatment is, after all, less difficult than that of irritable, though not intemperate individuals.

It is said and believed, that successive attacks of gonorrhœa become more mild and more amenable to treatment. This is far from universally correct. A gentleman is under my care, who contracted his first gonorrhœa at Cambridge. He laboured under discharge for six months. He lately experienced a second infection. The symptoms were exceedingly severe, and in spite of much care, though not of absolute precaution, hernia humoralis has resulted. A patient was lately in the hospital, from whom the following particulars were obtained. His first gonorrhœa had occurred three years before his admission; it lasted for six months—the second, two years before his admission; it lasted for two months—the third, sixteen months before his admission; it lasted for six weeks—the fourth, thirteen months before his admission; it lasted for five weeks—the fourth and last, seven months before his admission; it had lasted for those seven months, and for it he was admitted. I might mention many other similar facts. The surgeon should consider the patient's symptoms rather than the ordinal numbers of the gonorrhœa.

I have formerly remarked that itching in the urethra is a common symptom. It sometimes remains as a solitary feature, but more frequently it is observed as the cure is about to be effected. I always regard it, on this account, with a feeling of satisfaction. I suppose it depends on a condition of the vascular or nervous constitution of the part, similar to that which is observed on the subsidence of inflammation on the surface of the body. Itching is there a familiar precursor of recovery. The best remedy for it, as a symptom, appears to be purging and patience.

A little sticking, or a slight intumescence and redness of the orifice, not unfrequently remains after all discharge has disappeared. The patient is not safe. The slightest excess, exertion, or discontinuance of remedies, may be followed by return of the discharge.

The patient should never be considered cured till a fortnight has elapsed after every symptom has vanished. It is common for discharge to return after an absence of a week or ten days. I have seen the interval of apparent cure even longer than this. I invariably direct the patient to continue the means he has been using for three weeks after the cessation of the discharge, and not to return to wine, or beer, or venereal intercourse, for a longer period than that.

A patient not unfrequently inquires when he may venture to marry. I will state a case. A gentleman was under my care in September with acute gonorrhœa. The cure was apparently effected in a fortnight or three weeks, but slight sticking of the orifice remained for a week or two more. This patient was to have been married in November. I recommended him to wait till December. He did so, and neither his wife nor himself have experienced the slightest discharge. A gentleman is now under my care in whom the discharge has stopped for ten days. Supposing that it does not reappear, I have advised him to postpone the matrimonial connexion till February or March. This may be looked upon as over-prudent. A surgeon in the country recommended a patient, under circumstances of this kind, to drink much wine and have connexion often. This is over-rash. The fact is, that I lately saw a case in which the discharge had been absent for a month, and was re-induced by connexion with a woman perfectly clean.

